# The microbiota, the malarial parasite, and the mice—a three-sided relationship

**DOI:** 10.3389/fmicb.2025.1615846

**Published:** 2025-06-04

**Authors:** Shanli He, Yanwei Qi

**Affiliations:** ^1^The Second School of Clinical Medicine, Guangzhou Medical University, Guangzhou, China; ^2^Department of Pathogenic Biology and Immunology, School of Basic Medical Sciences, Guangzhou Medical University, Guangzhou, China

**Keywords:** *Plasmodium*, malaria, gut microbiota, mice, immune response, metabolic regulation

## Abstract

In recent years, the role of gut microbiota in modulating malaria susceptibility and infection progression has emerged as a pivotal focus in interdisciplinary research. While existing reviews have delineated mechanisms by which mosquito-associated gut microbiota regulate *Plasmodium* development, a systematic synthesis of the tripartite interplay among host gut microbiota, *Plasmodium* and host immunometabolic networks remains absent. Compared with previous studies predominantly focusing on single species or unitary mechanisms, this review fills the gap in cross-species integrated analysis of host-microbiota-pathogen interactions. By consolidating metagenomic, metabolomic, and immunological data, this review transitions from unitary mechanistic explanations to multi-omics-driven systematic analyses, demonstrating that murine microbiota suppresses *Plasmodium* proliferation through adaptive immune activation and metabolic product regulation. Meanwhile, *Plasmodium* infection induces decreased microbial diversity and functional pathway deviation in murine microbiota, exacerbating host immunometabolic imbalance. These advancements not only elucidate core biological principles governing “microbiota-host-pathogen” interactions but also transcend traditional pathogen-centric perspectives by pioneering precise intervention strategies based on microbiota homeostasis restoration. This provides theoretical foundation for developing microbiome-targeted precision prevention approaches, which will continue to make substantial contributions to malaria research.

## 1 Introduction

Malaria remains a significant global health challenge, with millions of cases and hundreds of thousands of deaths reported annually. According to World Malaria Report 2024 issued by the World Health Organization (WHO) highlights that global malaria cases reached approximately 263 million in 2023, reflecting an increase of 11 million compared to 2022. Malaria-related fatalities totaled 597,000 in the same year (WHO, 2024). The disease is caused by *Plasmodium* parasites, which are transmitted to humans through the bites of infected Anopheles mosquitoes. While substantial progress has been made in understanding the biology of the parasite and the host’s immune response, the role of the microbiota in this Multi-dimensional relation has only recently gained attention. Current malaria control strategies predominantly rely on antimalarial drugs, yet the global malaria landscape remains a critical public health challenge. Antimalarial drug resistance has emerged as a pivotal barrier to malaria elimination worldwide and a primary impediment to effective treatment and burden reduction. Innovative approaches leveraging mouse models have demonstrated promise, such as the development of lymph node-targeting nanovaccines (Howard et al., 2021), which enhance antigen-specific antibody responses to block parasite transmission. For example, nanoparticles delivering the mosquito midgut protein AnAPN1 induced potent transmission-blocking antibodies in mice, reducing *Plasmodium* oocyst formation in mosquitoes by targeting lymphatic drainage and optimizing adjuvant co-delivery. Additionally, studies on lactoferrin, an iron-binding protein, revealed its antiparasitic potential in mouse models by chelating iron essential for parasite survival (Anand, 2024). Native and nanoformulated lactoferrin disrupted *Plasmodium* growth by depriving parasites of iron and modulating host immune responses, highlighting its dual role in nutrient competition and immunoregulation. The microbiota, comprising bacteria, viruses, fungi, and other microorganisms, resides in various parts of the host’s body, particularly in the gut. This microbial community plays a crucial role in maintaining the host’s health by aiding in digestion, synthesizing essential nutrients, and modulating the immune system. Recent studies have shown that the microbiota can influence the host’s susceptibility to various infections, including malaria (Ippolito et al., 2018). Mouse models have been instrumental in dissecting these interactions, as demonstrated by comparative analyses of gut microbiota composition in genetically distinct mouse strains (Hugenholtz and de Vos, 2018). These studies revealed that host genetic background and rearing conditions significantly impact microbial diversity, affecting parasite burden and disease progression. For instance, specific bacterial taxa like *Lactobacillus* and *Bifidobacterium* in mouse gut microbiota were linked to reduced *Plasmodium* proliferation, underscoring the importance of standardized murine models to unravel microbiota-host–parasite dynamics. The mouse model has been instrumental in advancing our understanding of malaria. Mice share many physiological and immunological similarities with humans, making them valuable for studying the interactions between the microbiota, the malarial parasite, and the host. This review aims to provide a comprehensive overview of the three-sided relationship focusing on mainly three aspects: (1) the impact of the microbiota on malaria susceptibility, (2) the parasite’s manipulation of the host’s microbiota, and (3) the host’s immune response to both the microbiota and the parasite.

### 1.1 Rodent malaria parasites and host-specific interactions

Rodent malaria parasites (RMPs) serve as an essential experimental model, playing a crucial role in parasitology research and being widely applied in various aspects of malaria studies. These applications include parasite development within the host, drug resistance mechanisms, pathogenesis, and vaccine efficacy evaluation. *Plasmodium berghei* is a rodent malaria parasite widely used in malaria research, incapable of infecting humans, thereby exhibiting minimal biosafety risks during laboratory manipulations, simplified experimental procedures, highly reproducible results, and particular suitability for laboratories lacking infrastructure for high-risk pathogen handling. Although *P. falciparum* directly correlates with human malaria, its cultivation requires humanized mice or ethically complex human-mosquito cycle systems, alongside prolonged experimental timelines and high costs. The *P. berghei* model serves as an alternative system for rapid screening of candidate intervention strategies, followed by targeted validation in *P. falciparum* systems. *P. berghei* demonstrates high developmental efficiency and synchronization across all life cycle stages within mosquito vectors and murine hosts, enabling precise control of infection timepoints and suitability for investigating parasite–host-vector interaction mechanisms (Dehghan et al., 2018). During transmission of *P. berghei* via the bite of a female Anopheles mosquito, sporozoites are deposited into the murine host through the insect’s salivary glands, thereby initiating a new infection cycle. Genetic diversity exists between different strains of *P. berghei*, with the ANKA strain being a highly virulent *Plasmodium* capable of causing experimental cerebral malaria (ECM) and widely used to study how host factors affect ECM. The genome of *P. berghei* contains 14 chromosomes and has extensive genetic homology with human *Plasmodium* (Pattaradilokrat et al., 2022).

*Plasmodium yoelii*, a RMP widely utilized in murine infection models, serves as a cornerstone experimental system for malaria research. A landmark study by Swardson-Olver et al. (2002) demonstrated that *P. yoelii* employs the murine Duffy antigen receptor for chemokines (DARC) as a primary receptor for invading mature erythrocytes (normocytes). Using DARC-knockout mice, the authors revealed that normocyte invasion was drastically reduced in the absence of DARC, whereas reticulocytes remained susceptible, indicating a DARC-independent invasion pathway for immature erythrocytes. This study highlighted the species’ ability to exploit distinct receptors on different erythrocyte subsets, underscoring its utility in dissecting host–parasite interactions. The findings also revealed conservation of DARC-dependent invasion mechanisms between rodent and human malaria parasites, solidifying *P. yoelii* as a pivotal model for studying invasion biology and immune evasion. This species comprises multiple subspecies, which exhibit divergent virulence and host tropism, thereby providing a valuable resource for studying *Plasmodium* evolution.

*Plasmodium chabaudi*, a key representative of RMPs, with a life cycle similar to that of human *Plasmodium*. A comprehensive review by Stephens et al. (2012) highlights that *P. chabaudi* infection in mice recapitulates critical aspects of human malaria pathogenesis, including synchronized erythrocytic cycles, sequestration of infected red blood cells (iRBCs) in hepatic microvasculature, and induction of severe anemia with dyserythropoiesis. Infection with *P. chabaudi* in mice recapitulates the four major mechanisms underlying human malarial anemia, thereby establishing a severe anemia model. Compared to the *P. berghei* model—which often induces acute lethal cerebral malaria and shows significant pathological discrepancies from human cerebral malaria (CM)—*P. chabaudi*’s chronic disease progression is more suitable for investigating non-lethal pathologies (e.g., anemia). In contrast, the *P. yoelii* model, which primarily infects immature erythrocytes and lacks chronic infection characteristics, fails to simulate the long-term immune responses observed in human malaria.

While the three established RMPs, *P. berghei, P. yoelii*, and *P. chabaudi* have been developed as experimental models, *Plasmodium vinckei* has remained understudied due to limited genetic manipulation tools and phenotypic data. Carlton (2021) highlighted recent advances in understanding this fourth RMP species, which exhibits the broadest geographic distribution among the four RMPs. The authors emphasize that *P. vinckei*, containing four subspecies with marked genetic diversity, fills critical gaps of existing RMP models, such as the restricted genetic background of *P. berghei*, and emerges as an ideal system for investigating *Plasmodium* evolution, host adaptation, and multifunctional gene family dynamics.

### 1.2 Life cycle and pathogenesis of *Plasmodium* spp

*Plasmodium*, the causative agent of malaria, is a protozoan parasite with a complex life cycle that involves both vertebrate hosts and Anopheles mosquitoes. The life cycle of *Plasmodium* begins when an infected mosquito injects sporozoites into the host’s bloodstream during a blood meal. These sporozoites quickly travel to the liver, where they invade hepatocytes and undergo asexual replication exoerythrocytic schizogony, resulting in the formation of thousands of merozoites. The infected hepatocytes eventually rupture, releasing merozoites into the bloodstream, where they invade red blood cells (RBCs) and initiate the erythrocytic cycle. During the erythrocytic cycle, merozoites replicate within RBCs, leading to the formation of schizonts. The RBCs eventually rupture, releasing new merozoites that can infect additional RBCs. This cyclical destruction of RBCs is responsible for the clinical symptoms of malaria, including fever, anemia, and organ damage. Some merozoites differentiate into male and female gametocytes, which can be taken up by a mosquito during a blood meal. Within midgut of mosquito parasite undergoes sexual cycle and differentiate into ookiente, oocyst and finally sporozoite, thereby completing the cycle.

The pathogenesis of malaria is multifaceted and involves both parasite and host factors. The liver stage of *Plasmodium* development involves hepatocyte invasion, which may indirectly influence gut-liver axis signaling and systemic immune responses (Denny et al., 2019). The destruction of RBCs during the erythrocytic cycle leads to anemia and the release of toxic heme, which can cause oxidative stress and tissue damage, potentially disrupting gut barrier integrity and microbiota composition (Sriboonvorakul et al., 2023). The parasite’s interaction with the endothelium can result in the sequestration of infected RBCs in vital organs, such as the brain, leading to severe complications like cerebral malaria. The host’s immune response, while essential for controlling the infection, can also contribute to pathology. TNF-α and IL-1β directly amplify the inflammatory cascade by activating neutrophils, promoting the expression of endothelial cell adhesion molecules, and inducing the release of inflammatory mediators (Mehdi et al., 2025). The host’s immune response exemplifies a dual role: while pro-inflammatory mediators may induce immunopathology, they remain essential for controlling infection. This paradox is mechanistically validated by Anand et al. (2015b), who demonstrated that oral administration of alginate-enclosed, chitosan-conjugated lactoferrin nanocapsules in *P. berghei* infected mice significantly elevated pro-inflammatory cytokines TNF-α and IFN-γ, alongside increased reactive oxygen species (ROS) and nitric oxide (NO) production. This heightened inflammatory response was associated with reduced parasite load in spleen and liver tissues, highlighting the dual role of immune activation in both pathogen clearance and potential tissue damage. The study also showed that nanoformulated lactoferrin modulated gut microbiota-mediated immune signaling, balancing protective immunity against excessive inflammation. Excessive production of pro-inflammatory cytokines, such as TNF-α and IFN-γ, can cause systemic inflammation and tissue damage.

Understanding the biology and pathogenesis of *Plasmodium* is crucial for developing effective interventions against malaria. Through these investigations, which integrate epidemiological surveillance and molecular pathogenesis studies, a deeper mechanistic understanding of malaria transmission dynamics can be achieved, thus informing the development of next-generation antimalarial drugs and vaccine. Such advances are critical for disrupting parasite dissemination and mitigating the global burden of this disease. Concurrently, it is imperative to prioritize multidisciplinary collaborations and sustained funding initiatives to accelerate translational research in malaria therapeutics. By addressing these priorities, the international community can strengthen health systems and safeguard global public health security, particularly in endemic regions where socioeconomic disparities exacerbate vulnerability.

### 1.3 Composition of the gut microbiota in the host

The gut microbiota is composed of bacteria, viruses, fungi, archaea, and other microorganisms. Bacteria dominate this ecosystem, which is taxonomically classified into six major phyla: *Firmicutes*, *Bacteroidetes*, *Actinobacteria*, *Proteobacteria*, *Fusobacteria*, and *Verrucomicrobia*. Among these, *Firmicutes* and *Bacteroidetes* collectively account for over 90% of the total bacterial population, emerging as the predominant lineages (Hou et al., 2022).

Guo et al. (2022) conducted 16S rRNA sequencing on fecal samples from BALB/c and C57BL/6 J mice, which were categorized into disease-associated (DRA), immunodeficient (IDA), gene-edited (GED), and normal control groups. Their analysis revealed that host genetic background exerted the greatest influence on gut microbiota composition, with C57BL/6 J mice exhibiting greater microbial stability compared with BALB/c mice. Notably, *Bacteroidetes* and *Firmicutes*, two dominant phyla, displayed the most significant variations in microbial richness across the normal, IDA, GED, and DRA groups. Hildebrand et al. (2013) compared the microbiome composition of mice of different genotypes of mice and found that the more similar the genotypes between different strains of mice, the more similar the composition of their intestinal microbiome, so it is important to select strains of mice with identical genotypes for microbiome studies.

Xiao et al. (2015) found that mice of identical strains maintained distinct microbial signatures despite identical feed protocols across different laboratories. High-fat diets consistently elevated alpha diversity while reducing beta diversity in gut microbiota, driving microbial structural convergence and enriching genera such as *Clostridium* and *Butyrivibrio*. In contrast, low-fat diets correlated with higher abundances of *Prevotella* and *Bacteroides*. Laboratory environmental variables (e.g., bedding materials, operational protocols) further modified microbial metabolic functions. Additionally, strain-specific effects governed the colonization of particular taxa (e.g., *Akkermansia* and *Lactobacillus*). These multifactorial interactions underscore the necessity to strictly control housing variables in experimental designs to ensure comparability across experimental outcomes.

It is necessary to evaluate the structure of the gut microbiota of mice and to consider the influence of the gut microbiota of the experimental animals on the results of the experiments in future basic and clinical studies. Understanding the status of murine intestinal microbiome in mice and using high quality laboratory animals can significantly enhance the accuracy of experimental results. Recent investigations of intestinal microbiome have focused on their roles in diverse pathological conditions. In contrast, few investigations have been conducted on the bacterial community structure of representative mouse strains from the same environmental context excluding manufacturers and environmental facilities, and these studies are critical for addressing this knowledge gap and refining mechanistic interpretations of microbiome-host interactions.

### 1.4 Function of the gut microbiota in the host

The functional diversity of gut microbiota is directly influenced by its compositional structure (Hildebrand et al., 2013; Guo et al., 2022). Variations in microbiota composition and diversity induced by genetic backgrounds and rearing conditions lead to significant alterations in key functional modules, including SCFAs biosynthesis pathways and bile acid metabolism-associated gene abundance. These findings collectively indicate that structural characteristics of microbial communities constitute the foundation of their functional phenotypes. Increased microbiota α-diversity promotes *Clostridium genus* enrichment (Xiao et al., 2015), which enhances intestinal mucosal barrier function through IgA secretion stimulation and IL-12 signaling pathway activation, while simultaneously remodeling microbial ecology via induction of antimicrobial peptide REG3β expression (Stefka et al., 2014). Conversely, abnormal proliferation of *Proteobacteria* phylum exacerbates systemic inflammation through lipopolysaccharide (LPS) synthesis (Panzer and Lynch, 2015; Agus et al., 2016). This evidence demonstrates that microbial community composition serves as the biological basis driving host immune response and metabolic homeostasis remodeling. The following sections will systematically dissect how dynamic microbiota composition influences host physiology through immune activation and metabolic regulatory networks.

The gut microbiota plays an indispensable role in the development and regulation of host immune systems. Members of the phylum *Firmicutes* and *Bifidobacterium* spp. generate short-chain fatty acids (SCFAs) through dietary fiber fermentation, which are critical for maintaining intestinal homeostasis and systemic metabolic balance by enhancing mucosal barrier integrity, modulating immune responses, and suppressing inflammatory pathways. SCFAs exert anti-inflammatory effects through histone deacetylase (HDAC) inhibition, a process that promotes transcriptional activation of immunoregulatory genes and facilitates regulatory Treg cell differentiation while suppressing pro-inflammatory Th17 cell polarization. Furthermore, SCFAs activate G protein-coupled receptors (GPCRs), triggering signaling cascades that reduce pro-inflammatory cytokines and elevate anti-inflammatory mediators, thereby preserving epithelial barrier function. Intestinal epithelial cells and immune cells recognize microbial molecules through pattern recognition receptors (PRRs) and other mechanisms, initiating immune responses. Gut-associated lymphoid tissue (GALT) generates IgA that neutralizes pathogens and constrains the excessive proliferation of commensal bacteria, thereby establishing immune tolerance (Yoo et al., 2020).

Distinct microbial taxa directly orchestrate immune cell differentiation (Ivanov et al., 2022), as exemplified by *segmented filamentous bacteria* (SFB), which elicit Th17 cell polarization through epithelial adherence-triggered antigen presentation, thereby fortifying intestinal barrier integrity. Conversely, *Clostridium* spp. promote regulatory T cell (Treg) differentiation, while *Akkermansia muciniphila* drives T follicular helper cell (Tfh) commitment, collectively shaping immunological equilibrium. These mechanisms, operating at the interface of microbial ecology and host immunometabolism, establish the gut microbiota’s central role in maintaining mucosal homeostasis, combating enteric pathogens, and coordinating systemic immune responses.

Furthermore, the gut microbiota modulates host metabolism. Takeuchi et al. (2023) elucidated in murine models that gut microbiota associated with insulin sensitivity ameliorates host insulin resistance through multifactorial mechanisms. By generating SCFAs, these microbial communities activate intestinal L-cell secretion of glucagon-like peptide-1 (GLP-1), which enhances insulin signaling and glucose homeostasis. Concurrently, microbiota-mediated modulation of bile acid metabolism suppresses hepatic gluconeogenesis. Reduced circulating endotoxin levels further attenuate adipose tissue inflammation and macrophage M1 polarization, while strengthened expression of tight junction proteins in the intestinal epithelium preserves barrier integrity, mitigating systemic low-grade inflammation. Notably, specific commensals such as *A. muciniphila* upregulate peroxisome proliferator-activated receptor gamma (PPARγ) and mitochondrial biogenesis-related genes, a process that promotes white adipose tissue browning and energy expenditure, synergistically resolving glucolipid metabolic dysregulation.

Overall, the composition and function of the gut microbiota have a profound impact on the health and well-being of the host. Maintaining a healthy and diverse gut microbial community is essential for optimal physiological function.

## 2 Link between microbiota, host defense mechanism, and *Plasmodium*

### 2.1 Host types influence experimental reproducibility in malaria studies

Studies demonstrated that genetically similar mice from distinct commercial suppliers exhibited divergent gut microbiota profiles, which subsequently led to differential parasite burdens and mortality rates following *P. yoelii* infection (Villarino et al., 2016). Resistant mice, characterized by specific microbial compositions, displayed lower parasitic loads, reduced clinical severity, and elevated survival post-infection. In contrast, susceptible mice manifested heightened parasite burdens, severe clinical manifestations, and diminished survival. Mandal et al. (2020) revealed that spatiotemporal variations in gut microbiota of mice obtained from the same supplier at different timepoints significantly modulated host immune responses to *Plasmodium* infection. Early-stage microbiota facilitated rapid clearance of *P. yoelii*, whereas late-stage microbiota resulted in elevated peak parasitemia and delayed pathogen elimination.

Stough et al. (2016) observed significant differences in resistance patterns among C57BL/6 mice sourced from Taconic Biosciences versus Charles River Laboratories. Mice originating from Taconic exhibited markedly lower parasite burdens and accelerated recovery trajectories, while those acquired from Charles River Laboratories manifested higher parasitemia levels and delayed resolution kinetics. Taniguchi et al. (2015) revealed that *P. berghei* ANKA infection induces pathophysiological alterations in the intestinal mucosa, manifesting as epithelial apoptosis and compromised barrier function in C57BL/6 mice. These structural disruptions correlate with significant compositional changes in the gut microbiome, including a marked reduction in *Firmicutes* abundance and concomitant expansion of *Proteobacteria* taxa, hallmarks of microbial dysbiosis. Notably, this pathological phenotype was significantly attenuated in BALB/c mice.

### 2.2 *Plasmodium* species-driven divergence in gut microbial diversity

While the genetic and microbial characteristics of the host have been shown to play a pivotal role in malaria—related microbiota changes, the unique attributes of *Plasmodium* species also exert a profound impact on the composition and dynamics of the gut microbiota. Taniguchi et al. (2015) found that C57BL/6 mice infected with *P. berghei* ANKA exhibited intestinal pathological alterations concurrent with pronounced gut microbiota dysbiosis. This study represents the first experimental demonstration that *Plasmodium* infection directly induces microbiota dysbiosis, with the degree of microbial disruption closely linked to the severity of intestinal and cerebral pathological damage.

Even within the same mouse strain, intestinal microbiota exhibit marked divergence following infection with distinct *Plasmodium* species. Guan et al. (2022) reported that BALB/c mice infected with *P. yoelii* 17XL developed severe malaria accompanied by significant shifts in gut microbiota richness and diversity, with *Lactobacillaceae* emerging as the dominant family across all samples. Guan et al. (2023) further investigated the relationship between mild malaria and microbiota by analyzing gut microbial changes in BALB/c mice infected with the non-lethal strain *Py* 17XNL and evaluating parasitemia and survival rates at multiple post-infection timepoints. Significant alterations in microbiota richness and diversity were observed at days 9 and 15 post-infection, with near-complete restoration by day 28. These gut microbiota dynamics were closely associated with the progression of malaria pathology. Parasite presence activates host immune responses, disrupts microbial homeostasis, or engages in metabolic competition with commensal microbes for nutrients and ecological niches (Drew et al., 2021; Table 1. Differential Impacts of *Plasmodium* Species on Gut Microbiota in Murine Models).

**Table 1:** Differential impacts of *Plasmodium* species on gut microbiota in murine models.

*Plasmodium* species	Sample type	Host	Dynamic	Method of microbiome characterization	References
*P. yoelii*	Fecal samples	C57BL/6	Vendor-specific dysbiosis Increased fliC, ureABC, and nuo genes in susceptible mice	16S rRNA sequencing	Takeuchi et al. (2023)
*P. berghei* ANKA	Fecal samples	C57BL/6	Increased *Proteobacteria* and *Verrucomicrobia* Decreased *Firmicutes*	16S rRNA sequencing	Villarino et al. (2016)
*P. yoelii* 17XNL	Cecum tissue	BALB/c	Increased *Lactobacillus gasseri* at peak parasitemia Recovery of diversity post-infection	16S rRNA sequencing	Taniguchi et al. (2015)
*P. yoelii* 17XL	Fecal samples	BALB/c	Reduced alpha diversity Increased beta diversity post-infection	16S rRNA sequencing	Stough et al. (2016)
*P. berghei* ANKA	Fecal samples	C57BL/6	Healthy Stage: Dominant OTU265 (*Lactobacillaceae*) Infection Stage (8 dpi): OTU265 decreased, OTU147 (*Enterobacteriaceae*) increased significantly Cure Stage (9 days post-cure): OTU234 (*Bacteroidales_S24*) became dominant, microbiota failed to recover to pre-infection state	16S rRNA sequencing	Fan et al. (2019)
*P. berghei* ANKA	Fecal samples	C57BL/6 J	Day1-6 post-infection: Progressive divergence from controls Key Changes: Increased *Akkermansia*, *Alistipes*, and *Alloprevotella*; decreased *Dubosiella* and *Marvinbryantia* Day 6: Cecal weight lose, small intestine length increase; microbiota remained distinct from controls	16S rRNA sequencing	Knowler et al. (2023)
*P. yoelii*	Colorectal contents and tissues	BALB/c	Baseline (0%): High *Rikenellaceae*, *Sutterella* Rising (10%): Emergence of *Moryella, Erysipelotrichaceae* spp. Peak: *Lachnospiraceae* FCS020 increase, *Sutterella* decrease; T helper 17 (Th17) immune activation Recovery (0%): Microbiota partially recovered but retained elevated *Eubacterium plexicaudatum* and *Ruminococcus*	Full-length 16S rRNA sequencing, RNA transcriptome analyses	Yawen et al. (2022)

#### 2.2.1 *Plasmodium* impairs intestinal barrier integrity and provokes inflammatory responses

The intestinal barrier, functioning as a critical interface connecting the luminal environment and the host immune system, maintains decisive importance for both local and systemic health through its structural integrity. The gut microbiota plays a pivotal role in maintaining intestinal barrier integrity and mucosal homeostasis (Kim et al., 2013; Alam and Neish, 2018). Under physiological conditions, this barrier orchestrates the equilibrium between the substantial antigenic load within the intestinal lumen and local immune surveillance, simultaneously sustaining tolerance to common antigens while mounting rapid responses against pathogenic invasions. When intestinal barrier integrity is compromised or functionally impaired, the resulting “leaky gut” phenotype permits luminal bacteria and antigens to translocate across the epithelial barrier, triggering localized and systemic inflammatory responses (Neurath et al., 2025).

Brabin et al. (2019) discovered that *Plasmodium* upregulates inflammatory mediators (e.g., C-reactive protein) and hepcidin expression, suppressing intestinal iron absorption. This leads to iron accumulation in the gut, which alters gut microbiota composition, promotes *Salmonella* proliferation, and induces LPS release, ultimately causing intestinal barrier disruption and localized inflammation. Concurrently, elevated hepcidin impairs macrophage bactericidal capacity, exacerbating intestinal infections and systemic inflammation. Mooney et al. (2015) observed significant compositional shifts in the gut microbiota of *P. yoelii*-infected mice, characterized by reduced *Firmicutes* abundance and a relative increase in *Bacteroidetes*. This dysbiosis diminished host colonization resistance against *Salmonella Typhimurium*, not only elevating susceptibility to *Salmonella* infection by 34-fold in malaria-infected mice but also impairing resistance to other pathogenic bacteria.

Furthermore, Wright et al. (2023) demonstrated in a human malaria model that pregnant women infected with *P. falciparum* exhibited significantly elevated plasma concentrations of intestinal permeability biomarkers, which positively correlated with parasite burden. Sequestration of infected erythrocytes in intestinal microvasculature triggers localized ischemia, increasing intestinal permeability. Concurrently, reduced bioavailability of L-arginine, which is critical for maintaining intestinal tight junctions and mucus barrier, further compromises barrier integrity. In a mosquito model, Cardoso-Jaime and Dimopoulos (2025) revealed that phagocytic hemocytes of *Anopheles gambiae* do not directly suppress *P. falciparum* infection but instead facilitate its early colonization in mosquitoes by preserving midgut epithelial integrity. This finding challenges the traditional view of “mosquito immune systems universally antagonizing *Plasmodium*” highlighting the dual role of mosquito immunity during parasite infection.

#### 2.2.2 Metabolite competition and nutrient deprivation

*Plasmodium* infection not only reshapes gut microbiota through physical barrier disruption but also exacerbates microbial dysbiosis via metabolic resource competition. This metabolic exclusion effect likely impairs microbiota-mediated colonization resistance and accelerates host pathological progression. Ramírez-Carrillo et al. (2020) demonstrated that parasites can modulate diverse host behaviors by altering physiological pathways such as hormone and neurotransmitter signaling. These behavioral shifts represent outcomes of complex interactive networks between hosts and their microbiota. Certain gut bacteria have been found to suppress *Plasmodium* growth and development through direct competition or production of antimicrobial compounds. Villarino et al. (2016) identified enriched *Lactobacillus* and *Bifidobacterium* populations in resistant mice, which compete with *Plasmodium* for nutrient resources and host cell adhesion sites. This resource competition mechanism contributes to suppression of *Plasmodium* proliferation and infection severity (Spragge et al., 2023). In human malaria models, Yooseph et al. (2015b) observed that individuals with elevated gut abundances of *Bifidobacterium* and *Streptococcus* exhibited significantly reduced susceptibility to *P. falciparum* infection. Notably, this protective enterotype demonstrated cross-species conservation, aligning with the microbial profile identified by Villarino and colleagues in rodent malaria models (Villarino et al., 2016). Certain intestinal parasites exploit sugars derived from commensal bacterial breakdown of dietary fiber as their energy source, while simultaneously secreting metabolic toxins to inhibit the activity of beneficial bacteria. This metabolic competition not only compromises the nutritional contributions of commensal microbiota to the host but may also exacerbate pathological processes through disrupted host–microbe interactions (Kohl et al., 2014).

Although existing studies have preliminarily elucidated mechanisms of nutrient competition between parasites and host microbiota, the mechanisms underlying *Plasmodium* (an obligate intracellular parasite) competing with host microbiota for nutrients remain largely unexplored. Importantly, given that *Plasmodium* lives intracellularly during its exoerythrocytic and erythrocytic cycle, direct competition with gut microbiota for nutrients resources is unlikely. Drawing upon theoretical frameworks from other parasite studies, we hypothesize that *Plasmodium* may employ multiple strategies. First, *Plasmodium* might directly deplete critical metabolites (e.g., SCFAs, vitamins) produced by the host or commensal bacteria to sequester nutritional resources. Second, *Plasmodium* could inhibit commensal bacterial growth by modifying the host intestinal environment (e.g., pH, oxygen levels), thereby reducing their metabolite production. Additionally, *Plasmodium* may modulate host immune responses to indirectly alter the community structure of commensal microbiota, consequently shifting the distribution of metabolites.

#### 2.2.3 *Plasmodium* compromises microbiota-mediated immune or metabolic functions

Beyond metabolic competition, *Plasmodium* may further impair host defenses by interfering with microbiota-mediated immune or metabolic regulatory networks, thereby creating a self-reinforcing cycle of microbial dysbiosis and immunosuppression. Severe malaria infection disrupts gut-liver axis homeostasis (Denny et al., 2019), inducing dramatic alterations in gut microbiota composition and function, including enhanced bacterial motility, amino acid metabolic capacity, and infiltration of proinflammatory innate immune cells into intestinal mucosa, thereby exacerbating inflammatory responses. Liver damage and bile acid metabolic dysregulation further perturb microbiome structure. Severe infection-induced microbial dysbiosis may elevate intestinal bacteremia risk, highlighting the dual role of gut microbiota in malarial pathology as both defense modulator and disease accelerator.

Parasitic infection disrupts gut microbiota composition by triggering host inflammatory responses, leading to a reduction in beneficial commensals and impairing their immunomodulatory functions via metabolites such as SCFAs. Concurrently, pathogens deplete nutrients competitively and interfere with microbiota-derived immune-stimulating signals (e.g., MAMPs), further suppressing antimicrobial peptide secretion and immune cell activity, ultimately compromising host immunometabolic defenses (Khosravi and Mazmanian, 2013; Ezenwa, 2016; Hoarau et al., 2020). Moreover, coinfections may drive immune resource competition and host immune system overactivation, exacerbating pathological responses, while parasite-microbe antagonistic or synergistic interactions can remodel the host metabolic environment (Frisan, 2021).

#### 2.2.4 Gut iron homeostasis and parasite-nutrient competition

##### 2.2.4.1 Lactoferrin-mediated nutritional immunity

The availability of iron in the intestinal lumen represents a critical axis in host–parasite interactions, influencing microbial ecology and parasite virulence through nutrient competition and immune modulation. Yilmaz and Li (2018) reviewed how iron availability shapes gut microbiota composition, demonstrating that lactoferrin (Lf), a key iron-sequestering protein, limits pathogen growth by chelating iron in iron-restricted environments and modulates mucosal immune responses via TLR signaling. This dual role positions Lf as a central mediator in “nutritional immunity,” where iron restriction impedes parasite proliferation while supporting host defense mechanisms. Anand (2024) explored the antiparasitic effects of Lf across different iron-saturated forms, showing that apo-lactoferrin (iron-free) disrupts *Plasmodium* and *Toxoplasma* growth by depriving parasites of essential iron, whereas holo-lactoferrin (iron-bound) induces reactive oxygen species (ROS) in macrophages to enhance parasite clearance. These mechanisms highlight the importance of iron saturation status in Lf’s efficacy, with iron-deprived forms directly limiting parasite nutrition and iron-bound forms augmenting immune-mediated killing.

Clinical implications of iron-lactoferrin interactions are underscored by Stoltzfus (2012), who addressed the dilemma of iron supplementation in malaria-endemic regions. While excessive iron can exacerbate infection risk by promoting pathogen growth, Lf-based interventions offer a targeted approach to balance host iron needs and infection control. By integrating lifecycle strategies—such as prenatal iron management and low-dose formulations—Lf minimizes adverse effects while addressing iron deficiency, a common comorbidity in malaria-affected populations. Anand et al. (2015a) further demonstrated that Lf modulates erythrocyte and macrophage iron metabolism, inhibiting *Plasmodium* invasion by chelating labile iron and upregulating Toll-like receptors to enhance immune recognition. *In vitro* studies showed that iron-saturated Lf increased ROS production in macrophages, accelerating parasite degradation, while apo-Lf reduced erythrocyte susceptibility to invasion, highlighting its role in both direct parasite inhibition and immune activation.

##### 2.2.4.2 Therapeutic applications of lactoferrin and nanoformulations

Nanotechnology has expanded Lf’s therapeutic potential, as shown by Anand (2024), where nanoformulated Lf improved bioavailability and targeted intracellular parasites like *Toxoplasma* by modulating Th1-type immune responses (e.g., IFN-γ secretion) (Anand et al., 2015c). This approach minimizes systemic toxicity while enhancing local iron sequestration in infected tissues, offering a promising strategy for overcoming drug resistance. Conversely, Murr et al. (2021) linked moderate malnutrition to gut iron dyshomeostasis, revealing that iron deficiency exacerbates intestinal permeability and mucosal damage during *Plasmodium* infection. Lf intervention restored iron-regulated pathways (e.g., hepcidin-ferroportin axis), mitigating epithelial injury and preserving gut barrier function-critical for limiting parasite dissemination in nutritionally compromised hosts. Together, these findings establish gut iron dynamics as a pivotal node in host–parasite interactions, where Lf-mediated iron regulation represents a dual-edged sword: depriving pathogens of essential nutrients while bolstering host immune effector functions.

### 2.3 Microbial-immune interplay orchestrates antimalarial defense

The compositional shifts in gut microbiota represent adaptive restructuring rather than simple dysbiosis, through integrated multi-omics analyses, Fan et al. (2019) identified specific operational taxonomic units (OTUs) associated with malaria pathogenesis and convalescence. Their findings highlighted substantial microbiome remodeling during infection, with persistent alterations that failed to revert to baseline composition post-recovery. This dynamic reorganization emerged from tripartite interactions involving direct parasitic manipulation, immunological modulation and environmental perturbations including antibiotic exposure.

Intestinal cells including enterocytes, goblet cells, Paneth cells, and tuft cells establish a physical barrier via tight junctions, preventing pathogen invasion (Neurath et al., 2025). Goblet cells produce mucus to trap pathogens (McCauley and Guasch, 2015), while Paneth cells release antimicrobial molecules including lysozymes and defensins while supporting intestinal stem cell maintenance (Clevers and Bevins, 2013). Tuft cells initiate anti-parasite immunity through IL-25 secretion, a process dependent on the transcription factor Pou2f3 (Gerbe et al., 2016; Howitt et al., 2016). During parasitic infection, gut cells detect pathogen-derived signals through pattern recognition receptors such as TLRs, triggering alarmins like IL-25 to activate Th2-type immune responses. This stimulates enhanced mucus secretion, intestinal fluid leakage, and amplified smooth muscle contractions, collectively forming a secretory-sweeping mechanism for parasite expulsion. Mast cell-derived proteases simultaneously degrade tight junctions to promote luminal fluid flow and synergize with muscular contractions to eliminate parasites (Maizels et al., 2012).

**Figure 1:**
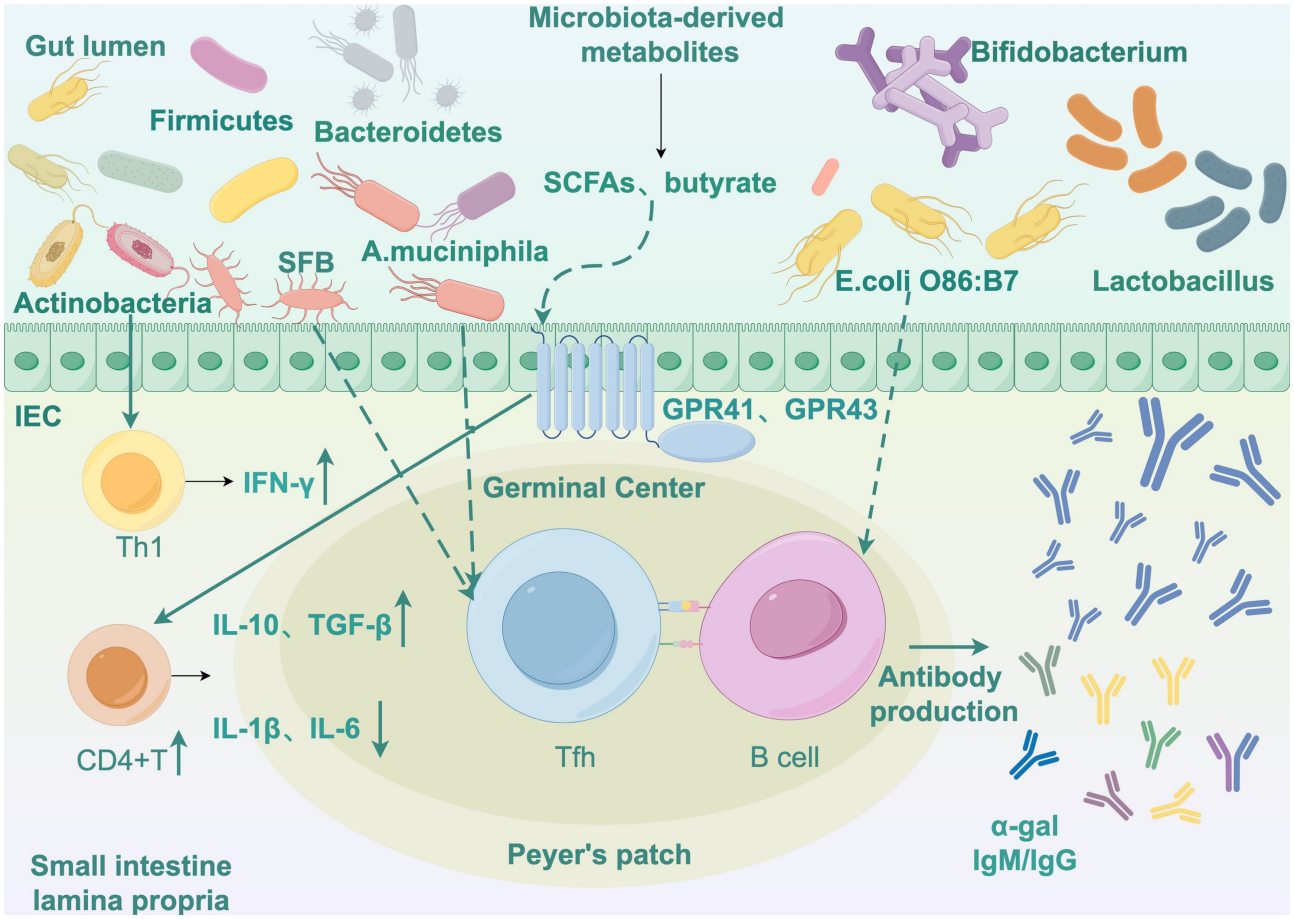
Interaction network of gut microbiota, host immunity, and *Plasmodium*. Draw by Figdraw. IEC, Intestinal epithelial cells. SFB, Segmented filamentous bacteria; *A. muciniphila*, *Akkermansia muciniphila*; SCFAs, Short-chain fatty acids; *E. colli, Escherichia coli*; Tfh, T follicular helper; GC Bcell, Germinal center cell; IFN-γ, Interferon gamma; IL-1β, Interleukin 1 beta; IL-6, Interleukin 6; IL-10, Interleukin 10; TGF-β, Transforming growth factor beta.

The resolution of malaria infection and the development of adaptive immunity depend critically on host T follicular helper (Tfh) cell and germinal center (GC) B cell differentiation and function (Figueiredo et al., 2017; Pérez-Mazliah et al., 2017), serving as pivotal determinants of disease severity and long-term immunological memory. These immunological mechanisms collectively provide a theoretical framework for microbiota-based interventions to enhance malaria control strategies (Figure 1).

#### 2.3.1 Microbiota-modulated humoral immunity

Divergent gut microbiota compositions elicit distinct malaria infection outcomes in mice, with low-parasite burden animals exhibiting increased GC-B cell numbers, elevated parasite-specific antibody titres, preserved GC structural integrity, and enhanced antigen-specific humoral responses. Conversely, susceptible mice exhibit early disruption of splenic GC architecture, compromising T-B cell interactions and functional maturation. The microbiota-driven resistance-associated B cell receptor (BCR) repertoire exhibits differential signaling patterns, with gut microbial communities modulating GC-mediated protective immunity against hyperparasitaemia. Such resistance confers protection against lethal malaria infections (Waide et al., 2020) through maintenance of GC reactions that suppress parasitemia peaks. Notably, specific gut commensals like SFB promote Tfh cell differentiation through intestinal lymphoid tissue homing, subsequently activating GC B cells (Teng et al., 2016) to produce high-affinity, polyclonal pathogen-specific antibodies. This microbiota driven humoral augmentation not only controls primary infection parasite loads but also establishes long-lived memory B cell pools conferring heterologous cross-protection against divergent malaria parasites (Figure 1).

Gut microbiota additionally modulates immune responses through metabolic products and cytokine regulation, which reciprocally influence T and B cell function to enhance malaria immunity (Pérez-Mazliah et al., 2015). Yilmaz et al. (2014) elucidated a protective mechanism involving microbiota-induced anti-α-gal antibody production, where *Escherichia coli* O86: B7 expressing α-gal antigens stimulate B cell production of α-gal IgM/IgG antibodies. These antibodies directly neutralize sporozoites, inhibiting their hepatocyte invasion and migratory pathways from necrotic skin tissues.

Although gut bacteria generally modulate host immunity to reduce *Plasmodium* infection severity, specific bacterial taxa may exacerbate severe malaria. Mandal et al. (2023) identified *Bacteroides* as a key genus driving susceptibility to severe malaria. In animal models, malaria-resistant mice developed hyperparasitemia after oral gavage with *Bacteroides fragilis* isolated from susceptible mice. Correspondingly, Ugandan children with severe malarial anemia exhibited significantly higher *Bacteroides* abundance compared to asymptomatic *P. falciparum*-infected children. Mechanistically, *Bacteroides* may suppress splenic germinal center responses via capsular polysaccharide or propionate production, reducing antimalarial antibody generation, or induce regulatory T cells (Tregs) to inhibit effector immune responses. Therefore, targeting intestinal *Bacteroides* (e.g., through antibiotics, probiotics, or dietary interventions) may reduce severe malaria risk. Key protective and pathogenic bacterial taxa identified in murine and human models are summarized in Table 2, highlighting their roles in modulating immune cell differentiation and inflammatory responses during *Plasmodium* infection.

**Table 2:** Key bacterial strains involved in inflammatory responses during malaria infection.

Bacterial strain	Host model	Inflammatory response	Key mechanisms	References
*Lactobacillus* spp.	Murine (C57BL/6, BALB/c)	Reduced parasite burden; enhanced Th1-type immunity (e.g., IFN-γ, TNF-α elevation)	Competitive nutrient acquisition; modulation of gut barrier integrity; induction of antimicrobial peptides	Villarino et al. (2016)
*Bifidobacterium* spp.	Murine (C57BL/6, BALB/c)	Suppressed *Plasmodium* proliferation; increased IgA secretion and Treg cell differentiation	SCFA-mediated inhibition of pro-inflammatory pathways; enhancement of mucosal barrier function	Villarino et al. (2016)
*Bacteroides fragilis*	Murine (C57BL/6)	Exacerbated severity; increased pro-inflammatory cytokines (IL-6, IL-1β) in susceptible mice	Suppression of germinal center responses; induction of regulatory T cells (Tregs) via capsular polysaccharides	Mandal et al. (2023)
*Akkermansia muciniphila*	Pregnant Swiss Webster mice	Reduced parasitemia; Th1 polarization (IFN-γ upregulation); improved fetal survival	Modulation of placental immune environment; inhibition of inflammatory cytokine IL-6	Morffy Smith et al. (2019)
*Enterobacteriaceae*	C57BL/6 mice (*P. berghei* ANKA infection)	Dysbiosis-associated gut permeability; increased LPS-induced systemic inflammation	LPS translocation activates TLR4 signaling, inducing excessive TNF-α and IL-1β; depletes protective metabolites (e.g., butyrate)	Taniguchi et al. (2015)
*Proteobacteria phylum*	C57BL/6 mice (*P. berghei* ANKA infection)	Increased abundance during dysbiosis; correlated with epithelial apoptosis and barrier dysfunction	LPS-mediated activation of TLR4/NF-κB pathway; induction of systemic inflammation (TNF-α, IL-6)	Taniguchi et al. (2015)
*Lactobacillus gasseri*	BALB/c mice (*P. yoelii* 17XNL infection)	Transient dominance during peak parasitemia; accelerated post-infection recovery	Metabolic competition for nutrients; production of short-chain fatty acids (SCFAs) to modulate immune homeostasis	Guan et al. (2023)
*Moryella* spp.	BALB/c mice (*P. yoelii* infection)	Early-stage Th1/Th2 polarization; increased cytokine production (IL-12, IFN-γ)	Activation of T cell receptor signaling; regulation of CD4 + T cell differentiation	Yawen et al. (2022)
*Ruminiclostridium* spp.	BALB/c mice (*P. yoelii* infection)	Inhibitory effect on Th17 cell differentiation during late infection stages	Modulation of gut microbial metabolism; suppression of pro-inflammatory pathways	Yawen et al. (2022)
*Bifidobacterium adolescentis*	Human (*P. falciparum* infection)	Negative correlation with infection susceptibility; enhanced mucosal IgA and anti-inflammatory cytokines	Production of antimicrobial metabolites; modulation of gut-liver axis signaling	Yooseph et al. (2015a)
*Escherichia coli O86: B7*	BALB/c mice	Inhibits *Plasmodium* sporozoite invasion of hepatocytes	Stimulates anti-α-gal IgM/IgG antibodies that neutralize sporozoites	Yilmaz et al. (2014)

#### 2.3.2 Microbiota-dependent cellular immune regulation

The gut microbiota not only enhances humoral immune responses in mice but also modulates cellular immunity through stage-specific regulation of T cell differentiation and immune receptor signaling during malaria infection. Yawen et al. (2022) revealed dynamic gut microbiota alterations closely associated with host immune responses during *P. yoelii* infection. Specific microbial communities at distinct infection stages (ascending phase, peak phase, convalescence phase) differentially regulate CD4 + T cell differentiation, early-stage *Moryella* spp. and *Erysipelotrichaceae* members activated Th1/Th2 polarization via positive regulation, whereas *Ruminiclostridium* spp. exhibited inhibitory effects. During later stages, Th17 cell differentiation correlated with increased *Peptococcaceae* and *Lachnospiraceae* abundances alongside decreased *Bacteroidales* BS11 populations. Moreover, persistent activation of TCR and BCR signaling pathways exhibited positive correlations with *Bacteroidales* BS11 and *Moryella*-dominated communities, suggesting microbiota-mediated regulation of T/B cell receptor signaling in host defense.

During malaria infection, gut microbiota additionally modulates the production of proinflammatory and anti-inflammatory cytokines. Appropriate cytokine regulation is critical for balancing effective parasite clearance with minimal tissue damage caused by excessive inflammation. Bamgbose et al. (2021) highlighted that microbiota-derived metabolites maintain immune homeostasis by promoting anti-inflammatory cytokines (e.g., IL-10, TGF-β) while suppressing proinflammatory mediators (e.g., IL-1β, IL-6), alleviating hyperinflammatory responses. Walker et al. (2021) demonstrated that *P. yoelii* infection activates host inflammatory pathways to upregulate TNF-α signaling, downregulate gastric acid secretion-related genes, reduce gastrin expression while increasing somatostatin levels, thereby impairing gastric acid barrier function. This impairment enhances gastric survival of enteric pathogens (such as *Salmonella*) and promotes their intestinal colonization (Figure 1).

The Th1/Th2 response balance is critical for infection resistance. Gut microbiota-driven immune regulation against parasites has been observed not only in *Plasmodium* models but also in *Entamoeba histolytica* and *Toxoplasma gondii* infections. Macrophages detect *E. histolytica* virulence factors via Toll-like receptors (Ivory et al., 2008; Yarovinsky, 2014), while dendritic cells activated by TLRs release pro-inflammatory cytokines and upregulate co-stimulatory molecules CD40, CD80, and CD86 to initiate adaptive immunity (Vivanco-Cid et al., 2007). NK and NKT cells activate neutrophils/macrophages through TNF-α and IFN-γ secretion, cytokines essential for hepatic defense against amebic liver abscesses, whose deficiency exacerbates pathology (Lotter et al., 2009). In adaptive immunity, Th1-polarized IFN-γ responses confer protection, as IFN-γ deficiency induces cerebral necrosis and acute inflammation, whereas Th2-biased IL-4 elevation correlates with invasive amebiasis (Guo et al., 2008). Notably, IL-4 exerts unique protective effects in *T. gondii* infection by enhancing IFN-γ production via Th2 polarization, contrary to its pathogenic role in most protozoan infections (Shaw et al., 2009). Table 2 provides a comprehensive overview of bacterial strains associated with altered inflammatory outcomes, including their host-specific effects on cytokine profiles and immune signaling pathways.

#### 2.3.3 Microbiota-mediated immune protection in pregnancy

The gut microbiota not only modulates malaria pathogenesis severity but also exerts indirect effects on pregnancy outcomes through immune modulation in the mother and placental function. Morffy Smith et al. (2019) provided critical insights by demonstrating that pregnant Swiss Webster mice receiving fecal microbiota transplantation (FMT) from malaria-resistant donors exhibited reduced parasite burdens, alleviated anemia, and diminished gestational weight loss. These improvements correlated with enhanced fetal and offspring survival rates at birth, alongside superior birth weights. Specific microbial taxa including *A. muciniphila*, *Allobaculum*, and select *Lactobacillus* species exhibited negative correlations with parasitemia, potentially mediating malaria suppression through Th1 immune polarization (e.g., upregulating IFN-γ expression). Conversely, high parasite loads triggered placental monocyte infiltration, increased hemoglobin deposition, and reduced vascular density-pathological changes linked to intrauterine growth restriction and neonatal mortality. Furthermore, gut microbiota modulated local immune environments (e.g., IL-10 levels) to regulate placental inflammatory responses. These findings collectively suggest that gut microbiota can override the influence of genetic factors in shaping malaria severity through immunoregulatory mechanisms, offering theoretical foundations for microbiota-based interventions to improve pregnancy outcomes during malaria. As outlined in Table 2, strains such as *Akkermansia muciniphila* exert protective effects via Th1 polarization and suppression of placental inflammation, aligning with findings from murine pregnancy models.

#### 2.3.4 Lung microbiota and malaria-associated complications

Emerging evidence extends the microbial-immune paradigm beyond the gut-liver axis to encompass pulmonary complications. Recent studies have uncovered critical interactions between lung microbiota and malaria pathogenesis. In experimental malaria-associated acute respiratory distress syndrome (MA-ARDS), dysbiosis of the lung microbiota is strongly correlated with disease severity. The study used two distinct rodent models (C57BL/6J mice infected with *P. berghei* K173 and DBA/2 mice infected with *P. berghei* ANKA) to show that *P. berghei* ANKA infection in mice induced a significant increase in bacterial load and altered microbial composition in the lungs, dominated by *Proteobacteria* and *Bacteroidetes* families. By comparing germ-free and specific pathogen-free mice, it was found that host microbiota is a contributing factor for MA-ARDS. This dysbiosis was driven by IL-10 mediated immunosuppression from T cells, which compromised bacterial clearance and exacerbated pulmonary inflammation. Notably, antibiotic treatment with linezolid reduced lung bacterial burden and improved survival rates in MA-ARDS models, demonstrating the therapeutic potential of microbiota modulation (Mukherjee et al., 2022). Furthermore, in the study of MA-ARDS, proteomic analyses used multiple serum protein extraction methods (direct digestion, TCA precipitation, and DTT/ACN treatment) to comprehensively cover the serum proteome. The results revealed that acute-phase response proteins (e.g., SAA1, CRP) were upregulated. Orthogonal validation was carried out, but it was unable to differentiate these proteins between mice with the development of ARDS and hyperparasitemia. These proteins may potentially interact with lung microbiota to drive alveolar-capillary barrier dysfunction (Rosa-Fernandes et al., 2025).

### 2.4 Microbiota-mediated host-microbial immune-metabolic crosstalk in erythrocytic-stage infection

The gut microbiota plays a critical role in nutritional metabolism and nutrient availability, with its compositional changes during malaria infection potentially altering nutrient accessibility to influence parasite growth and survival. Probiotics such as *Lactobacillus* spp. enhance serum nitric oxide (NO) levels to suppress intraerythrocytic parasite proliferation, thereby reducing parasitemia. Hepatic-derived bile acids suppress pathogenic bacterial growth by inducing antimicrobial peptide expression, thereby maintaining gut microbiota homeostasis, while intestinal microbes modulate hepatic bile acid and lipid synthesis, establishing a hepatic-intestinal metabolic crosstalk network (Tripathi et al., 2018). Disruption of this axis directly or indirectly impacts hepatic-stage *Plasmodium* infection (Mancio-Silva et al., 2017; Zuzarte-Luís et al., 2017). Liver damage is particularly pronounced in severe malaria, accompanied by alterations in bile acid metabolism that further destabilize gut microbial homeostasis (Denny et al., 2019).

While SCFAs exert pleiotropic effects on host immunity, Chakravarty et al. (2019) revealed that the regulatory role of gut microbiota in malaria severity exhibits no direct correlation with fecal SCFA levels. Gut microbiota may metabolize antimalarial drugs, altering their bioavailability and efficacy. The presence or absence of specific microbial strains could either enhance or suppress drug metabolic rates, thereby affecting therapeutic outcomes against malaria. Studies have shown that different types of antibiotics exhibit significant variations in their effects on gut microbiota. Although the specific patterns of species depletion depend on microbial community composition and antibiotic types, antibiotic treatment is consistently associated with marked reductions in the relative abundance of core taxa, including *Bacteroidetes*, *Firmicutes*, and *Actinobacteria*. This loss of diversity creates an ecological niche vacancy that facilitates colonization or overgrowth of opportunistic pathogens from the *Proteobacteria* phylum, contributing to various gastrointestinal disorders. These opportunistic pathogens often harbor transferable antibiotic resistance genes or possess intrinsic resilience traits such as sporulation, enabling their survival under antibiotic pressure (Fishbein et al., 2023).

In mosquito malaria models, Vinayagam et al. (2023) systematically reviewed that gut microbiota in mosquitoes directly suppress *Plasmodium* development by activating the immune deficiency IMD pathway and peptidoglycan recognition protein PGRP-mediated immune responses, thereby inducing thioester-containing protein TEP1 expression and antimicrobial peptide secretion. Concurrently, microbiota stimulate host-derived reactive oxygen species production to eliminate parasites while modulating microbial colonization, synthesize secondary metabolites targeting *Plasmodium* developmental stages, and regulate antioxidant enzymes such as catalase to mitigate oxidative stress, thereby preventing host tissue damage and mortality. This immune-metabolic synergy highlights microbiota-mediated dual suppression of *Plasmodium* infection via immunological and metabolic axes. These regulatory strategies exhibit cross-species conservation in arthropod vectors and mammalian hosts, though specific metabolites may vary due to microbiome compositional divergence.

### 2.5 Microbiota and antimalarial drug resistance

Current evidence (Wilson and Nicholson, 2017) indicates that structural variations in gut microbial communities regulate drug bioavailability and metabolic pathways via enzymatic transformations such as nitro-reduction and hydrolytic deconjugation, a phenomenon hypothesized to extend to antimalarial compound metabolism. Gut microbiota may metabolize antimalarial drugs such as artemisinin and chloroquine, altering their bioavailability and therapeutic efficacy. The presence or absence of specific bacterial strains may enhance or attenuate drug metabolic rates, thereby modulating treatment outcomes in *Plasmodium* infection.

Notably, certain antimalarial agents exhibit broad-spectrum antimicrobial properties. Zhang et al. (2022) first identified that *bacteriophage* Gp46 protein inhibits bacterial and *Plasmodium* growth by binding to the highly conserved HU protein, a critical DNA-binding protein in both organisms, through competitive occupation of its DNA-binding sites, thereby blocking HU-DNA interactions and inducing filamentous morphology due to impaired chromosome segregation. This dual antimicrobial-antimalarial activity suggests that some antimalarial drugs may disrupt gut commensal microbiota by suppressing specific strains or promoting resistance gene dissemination. Additionally, Dada et al. (2018) demonstrated that antibiotic treatments alter microbiota diversity in insecticide-resistant mosquitoes: vancomycin reduced resistance, whereas streptomycin and gentamicin enhanced resistance phenotypes. Resistance-exhibiting mosquitoes exhibited lower microbial diversity compared to susceptible counterparts, implying that antibiotic supplementation could modulate microbial load and diversity. Specific microbiota may contribute to resistance mechanisms via insecticide degradation or induction of detoxifying metabolic enzymes. This microbiota-resistance interplay highlights the potential of microbial-targeted strategies to reverse insecticide resistance.

Understanding the intricate interplay between gut microbiota, host defense mechanisms, and malaria pathogenesis is essential for identifying novel therapeutic targets and intervention strategies. Notably, this field remains under active investigation, necessitating further research to delineate the molecular pathways governing these complex interactions.

## 3 Problems and potential of treating malaria through modulating gut microbiota

### 3.1 Potential and problems of treating malaria through modulating gut microbiota

#### 3.1.1 Probiotic intervention

Villarino et al. (2016) investigated the role of gut microbiota in malaria pathogenesis, revealing that *Lactobacillus* and *Bifidobacterium* spp. confer protective immunity against malaria through competitive nutrient acquisition and host cell adhesion interference. Administration of yogurt containing these probiotics significantly reduced parasite burdens in susceptible mice, alleviating disease severity, thereby suggesting that microbiota modulation could serve as a preventive and therapeutic strategy against malaria. Waide and Schmidt (2020) explored the translational potential of gut microbiota manipulation in malaria control, highlighting the cost-effectiveness of probiotic interventions. These live microbial preparations can be mass-distributed through freeze-dried oral formulations without requiring cold-chain storage or professional medical supervision, making them particularly suitable for malaria-endemic regions with limited healthcare infrastructure. Mechanistically, gut microbiota enhance host adaptive immunity by promoting high-affinity antimalarial antibody production, while specific microbial taxa induce cross-protective antibodies that directly block malaria sporozoite hepatocyte invasion, a critical step overcoming parasite drug resistance. This strategic combination of microbiota engineering and vaccination could potentially enhance vaccine efficacy and longevity by synergizing microbiota-regulated immune responses with antigen-specific vaccination. The researchers proposed an immunological training hypothesis, positing that gut microbiota “train” monocytes and macrophages through epigenetic reprogramming, enabling more efficient parasite clearance. Consequently, the dynamic window of early infancy, when gut microbiota undergoes critical developmental changes, represents a unique therapeutic window for microbiota-based interventions to reduce severe malaria complications.

While probiotics offer benefits, they also present drawbacks and potential risks. Probiotic administration exhibits marked heterogeneity, with clinical efficacy fluctuating based on strain, dosage, and host factors (e.g., baseline microbiota, diet, genetic background). Some studies demonstrate limited or null efficacy against specific conditions (e.g., acute gastroenteritis, irritable bowel syndrome), with potential symptom prolongation. Prolonged or inappropriate use may disrupt natural microbiota recovery, suppress native microbial reconstitution, reduce α-diversity, and paradoxically elevate risks of pathogenic infections (e.g., *Clostridioides difficile*) or metabolic disorders. Vulnerable populations (preterm infants, immunocompromised individuals, critically ill patients) face severe complications like bacteremia, fungemia, or necrotizing enterocolitis (NEC). Furthermore, mucosal colonization capacity varies interindividually, with ~40% of individuals exhibiting “colonization resistance” leading to intervention failure. Successfully colonized strains may trigger paradoxical effects (e.g., inflammation promotion or tumorigenesis) via metabolites (e.g., bile salt hydrolases) or immune modulation. Regulatory gaps and commercial motives exacerbate safety uncertainties, as some products exhibit strain misidentification and product quality inconsistencies. Overall, probiotics are not universally safe solutions and require prudent application guided by personalized assessments (Suez et al., 2019).

#### 3.1.2 Fecal microbiota transplantation

Beyond probiotic development, FMT represents another therapeutic approach. To date, no evidence supports FMT for malaria treatment; its primary clinical application remains recurrent *Clostridioides difficile* infection (CDI) (Quraishi et al., 2017; Ianiro et al., 2018), where it demonstrates high safety and remarkable efficacy (85–90% cure rates). However, its translation to chronic non-communicable diseases faces substantial challenges, likely attributable to the relatively straightforward etiology of CDI, primarily driven by gut microbiota dysbiosis (Ling et al., 2014; Staley et al., 2016), whereas in complex diseases, gut microbiota constitutes only one component of multifactorial pathogenesis. FMT also carries potential risks of transmitting multidrug-resistant bacteria (e.g., *E. coli*) or pathogens (e.g., SARS-CoV-2), necessitating caution, particularly in immunocompromised populations. Consequently, FMT alone is unlikely to replicate its CDI success in complex diseases, requiring integration with complementary therapies and microbiota optimization to achieve clinical translation.

#### 3.1.3 Species-specific conversion bottleneck

Georgiadou et al. provided a significant breakthrough in linking rodent malaria to human malaria. In comparative transcriptomic analyses, *P. yoelii* 17XL-infected mice have emerged as a translational model that recapitulates key gene expression signatures of human severe malaria (Georgiadou et al., 2022). This murine model demonstrates transcriptional concordance with major human severe malaria syndromes, including hyperlactatemia and cerebral malaria, characterized by shared upregulation of neutrophil degranulation and myeloid leukocyte activation pathways. Mechanistically, *P. yoelii* 17XL infection induces hyperparasitemia, severe anemia, and hyperlactatemia comparable to human disease, alongside cerebral microvascular pathology marked by perivascular fibrinogen deposition and intravascular thrombus formation-features analogous to human cerebral malaria. These findings highlight the utility of *P. yoelii* 17XL mice for investigating conserved immune responses and pathogenic mechanisms, such as type I interferon signaling and neutrophil extracellular trap formation, which bridge rodent and human malaria pathogenesis. The model’s ability to mirror transcriptional programs associated with life-threatening human malaria phenotypes provides a robust framework for preclinical studies aimed at translating mechanistic insights into therapeutic strategies.

However, despite the high genomic concordance of the *P. yoelii* 17XL model with human cerebral malaria (HCM), other commonly used murine models (e.g., *P. berghei* ANKA-induced experimental cerebral malaria, ECM) exhibit significant limitations that necessitate cautious translational interpretation. A key limitation lies in the distinct mechanisms of parasite sequestration: unlike HCM, where parasitized red blood cells (pRBCs) intensely sequester within cerebral microvasculature, ECM in mice (e.g., *P. berghei* ANKA-infected CB57BL/6 or CBA mice) involves sequestration of parasite-infected leukocytes rather than pRBCs in the brain (White et al., 2010; Krishnan, 2017). This discrepancy underscores the divergent immunopathogenic pathways, as murine ECM relies heavily on CD8 + T cell-mediated vascular damage and inflammatory cytokine release (e.g., IL-33, perforin), whereas human CM involves pRBC adhesion to brain endothelium with less defined immune effector roles (Krishnan, 2017). This distinction is critical: while 92% of adjunctive interventions tested in murine models show efficacy, only 6% of such treatments have demonstrated benefit in human clinical trials. For example, anti-TNF antibody and dexamethasone, which ameliorate disease in mice, were ineffective or harmful in humans, underscoring the risk of translating murine findings to clinical settings. Nevertheless, these models remain essential for generating mechanistic insights that inform hypothesis-driven human research.

Furthermore, the discrepancies between murine and human models extend beyond mechanisms of *Plasmodium* infection. Hugenholtz and de Vos (2018) emphasized that although murine and human gut microbiota share dominance of *Bacteroidetes* and *Firmicutes* phyla, significant compositional and abundance differences exist. The murine gut is enriched with *Mucispirillum schaedleri* and SFB, which are minimally present in humans, whereas humans exhibit higher abundance of *A. muciniphila*. Metabolically, mice recycle microbial fermentation products (e.g., SCFAs) via coprophagic behavior, whereas human metabolite absorption depends on intestinal transit time and mucosal layer thickness. Immunologically, murine immune regulatory mechanisms remain unvalidated in humans, with low conservation of immune genes between species, leading to divergent inflammatory pathway responses (Krych et al., 2013; Nguyen et al., 2015). Furthermore, murine microbiota composition is strongly influenced by genetic backgrounds, suppliers, and housing conditions, potentially limiting extrapolation of experimental results to humans. These disparities underscore the necessity for cautious interpretation of murine models in human microbiota studies. Future research requires standardized protocols to enhance the reliability of evidence derived from rodent malaria models.

### 3.2 Future research directions of treating malaria through modulating gut microbiota

To address the potential risks of probiotics, future studies should prioritize establishing precision-guided and personalized probiotic application strategies, integrating multi-dimensional host factors (genetic background, gut microbiota profiles, dietary metabolism) to develop AI-driven colonization prediction models and screen next-generation probiotic strains with well-defined molecular mechanisms. Multi-center, large-scale randomized controlled trials (RCTs) must be advanced, harmonizing efficacy evaluation criteria and incorporating long-term safety monitoring (particularly in children, immunocompromised individuals, and critically ill patients) to elucidate probiotics long-term impacts on microbiota reconstitution, metabolic programming, and immune maturation. Simultaneously, leveraging single-cell sequencing, metabolomics, and organoid models can dissect the probiotic-host-microbiota tripartite interaction network, dismantling the “black-box” application paradigm to establish direct associations between strain functionality and clinical endpoints. Finally, regulatory frameworks require enhancement to standardize strain identity, dosage, and indication specifications, fostering interdisciplinary collaborations to accelerate evidence-based clinical translation (Waide and Schmidt, 2020).

Although the human and mouse gut microbiota share many taxonomic similarities, translational accuracy is hampered by notable variations in microbial quantity and composition. A possible method for more accurately simulating human gut ecology in experimental models is the use of humanized gnotobiotic mice that have been colonized with microbiota obtained from humans. To refine murine models, developing “humanized microbiota mice” by colonizing germ-free mice with fecal microbiota from healthy donors or malaria patients, combined with humanized immune systems, can better mimic human host–microbe interaction dynamics. Multi-center harmonized protocols should standardize diet formulations, housing conditions, and antibiotic pretreatment regimens, enabling cross-laboratory comparability of murine microbiota backgrounds through shared metagenomic databases. Introducing synthetic microbial consortia to precisely control colonization abundance of specific taxa (e.g., *Bacteroidetes*/*Firmicutes* ratios), alongside CRISPR-Cas9-mediated strain gene editing, will elucidate functional microbial mechanisms underlying *Plasmodium* inhibition.

Future directions in translational research should prioritize bridging insights from murine models with human clinical data through a two-pronged approach. First, validated pathways identified in humanized mouse models, such as those regulating liver-stage infection (e.g., EphA2-mediated hepatocyte invasion) or immune evasion mechanisms, should be corroborated using transcriptomic and proteomic datasets from malaria patients, particularly those with severe disease or treatment resistance (Vaughan et al., 2015; Minkah et al., 2018). Second, next-generation humanized mouse models must be developed to recapitulate human physiology more faithfully, integrating functional human hepatocytes (e.g., FRG huHep mice), erythropoiesis, and immune systems (HIS mice) to enable study of the complete *P. falciparum* life cycle, including hypnozoite persistence in *P. vivax* and sequestration-mediated pathology (Mikolajczak et al., 2015; Soulard et al., 2015). Such models should also incorporate human endothelial receptors (e.g., EPCR, ICAM1) to investigate tissue-specific sequestration and cerebral malaria pathogenesis, while dual-chimeric systems (HIS huHep) could resolve the role of human T cell subsets and antibody responses in vaccine-mediated protection (Huang et al., 2015; Li et al., 2016). Long-term, integrating these models with clinical omics data will accelerate translation of preclinical findings into novel therapeutics and vaccines.

For human studies, interventional clinical trials should evaluate the adjunctive efficacy of probiotics (e.g., *Lactobacillus*, *Bifidobacterium*) or microbiota-derived metabolites (SCFAs, bile acid derivatives) in enhancing antimalarial therapy, particularly focusing on improved treatment responses against artemisinin-resistant strains. Establishing malaria patient gut/blood microbiome cohorts with longitudinal sampling across infection phases (acute, chronic, post-treatment) will capture microbiota dynamics. Integrating host immune phenotypes and metabolomic profiles can identify diagnostic or prognostic microbial biomarkers.

Despite these challenges, the therapeutic potential of gut microbiota manipulation in malaria prevention continues to attract considerable interest. Future research will deepen our understanding of the host-microbiome-malaria axis through integrated multi-omics analyses, ultimately advancing the development of safe, efficacious microbiome-based interventions that complement existing malaria control frameworks.

## 4 Conclusion

Modern disease ecology proposes the “disease pyramid” model, transcending the traditional pathogen-centric paradigm to emphasize quadripartite dynamic interactions among host, parasite, environment, and microbiome. Within this framework, habitat disruption, host susceptibility, parasite pathogenicity, and microbiome stability collectively determine disease outcomes through direct or indirect pathways (Bernardo-Cravo et al., 2020). As a core component of the One Health concept, the microbiome exhibits tripartite critical properties, sensitivity to environmental perturbations and host health status (Fackelmann et al., 2021), reciprocal modulation of host immunity and disease progression (Kamada et al., 2013), and acceleration of pathogen evolution via horizontal gene transfer or mutations during dysbiosis, generating antibiotic resistance threats (Stecher et al., 2013; Wotzka et al., 2017).

This study systematically delineates the dynamic tripartite interaction network among gut microbiota, *Plasmodium*, and murine hosts, transcending prior research constraints limited to vector-borne microbiota or single-host models. It bridges the knowledge gap regarding mammalian host microbiota’s regulatory mechanisms throughout the *Plasmodium* life cycle. We pioneer the first cross-species elucidation of three core interaction hallmarks: (1) microbiota synergistically suppresses *Plasmodium* proliferation via immune activation and metabolic coordination; (2) *Plasmodium* infection destabilizes gut-liver axis homeostasis, driving microbiota functional pathway divergence; (3) host genetic background and microbial signatures collectively determine infection outcomes.

This study provides three pivotal advances: (1) Methodological Innovation. By integrating metagenomic, metabolomic, and immunologic datasets, we established the first multi-omics framework investigating *Plasmodium*-microbiota-mice interactions, overcoming limitations of single-mechanism studies. This enabled dynamic visualization of immune-metabolic pathway rewiring across infection progression. (2) Theoretical Advancement. We proposed the “microbiota-*Plasmodium* niche competition” hypothesis, demonstrating that *Lactobacillus* depletes heme iron (an essential parasite proliferation factor) to achieve nutrient deprivation. This mechanism was validated as evolutionarily conserved across rodent *Plasmodium* models. (3) Translational Breakthrough. FMT reduced placental inflammatory cytokine IL-6 levels and enhanced fetal survival in pregnant murine models, offering novel insights for clinical adjunctive therapy development.

Current research gaps primarily stem from incomplete understanding of parasite-microbiota interaction mechanisms. First, the precise molecular mechanisms by which parasites directly regulate host microbiota composition and diversity remain unclear, particularly the critical pathways through which pathogens actively modify microbial environments to enhance their survival and transmission. Second, existing studies are limited by incomplete elucidation of microbiota-modulating molecular mechanisms, especially insufficient evidence regarding microbiota-metabolite interactions with *Plasmodium* species, alongside unestablished standardized correction systems for individual variability’s impact on experimental outcomes. Current investigations predominantly rely on rodent models, lacking validation through human-derived data. Furthermore, systematic characterization of post-*Plasmodium* infection alterations in human gut/blood microbiota and their correlations with disease severity and treatment responses remains absent, constraining innovation in microbiota biomarker-based diagnostics and probiotic therapeutic strategies. Future investigations should prioritize developing microbiota gene-editing technologies to dissect functional microbial communities and exploring translational applications of probiotic interventions in malaria prevention and treatment. These advancements will catalyze a paradigm shift in malaria control from pathogen-centric approaches to integrated “microbiota-pathogen-host” regulatory frameworks.

## References

[ref1] Agus A. Denizot J. Thévenot J. Martinez-Medina M. Massier S. Sauvanet P.. (2016). Western diet induces a shift in microbiota composition enhancing susceptibility to adherent-Invasive *E. coli* infection and intestinal inflammation. Sci. Rep. 6:19032. doi: 10.1038/srep19032, PMID: 26742586 PMC4705701

[ref2] Alam A. Neish A. (2018). Role of gut microbiota in intestinal wound healing and barrier function. Tissue Barriers 6:1539595. doi: 10.1080/21688370.2018.1539595, PMID: 30404570 PMC6389125

[ref3] Anand N. (2024). Antiparasitic activity of the iron-containing milk protein lactoferrin and its potential derivatives against human intestinal and blood parasites. Front. Parasitol. 2:1330398. doi: 10.3389/fpara.2023.1330398, PMID: 39816822 PMC11731944

[ref4] Anand N. Kanwar R. K. Dubey M. L. Vahishta R. K. Sehgal R. Verma A. K.. (2015a). Effect of lactoferrin protein on red blood cells and macrophages: mechanism of parasite-host interaction. Drug Des. Devel. Ther. 9, 3821–3835. doi: 10.2147/DDDT.S77860, PMID: 26251568 PMC4524381

[ref5] Anand N. Kanwar R. K. Sehgal R. Kanwar J. R. (2015b). Antiparasitic and immunomodulatory potential of oral nanocapsules encapsulated lactoferrin protein against *plasmodium berghei*. Nanomedicine 11, 47–62. doi: 10.2217/nnm.15.181, PMID: 26654428

[ref6] Anand N. Sehgal R. Kanwar R. Dubey M. Vahishta R. Kanwar J. R. (2015c). Oral administration of encapsulated bovine lactoferrin protein nanocapsules against intracellular parasite *toxoplasma gondii*. Int. J. Nanomedicine 10, 6355–6369. doi: 10.2147/IJN.S85286 26504384 PMC4605239

[ref7] Bamgbose T. Anvikar A. R. Alberdi P. Abdullahi I. O. Inabo H. I. Bello M.. (2021). Functional food for the stimulation of the immune system against malaria. Probiot. Antimicrob. Proteins 13, 1254–1266. doi: 10.1007/s12602-021-09780-w, PMID: 33791994 PMC8012070

[ref8] Bernardo-Cravo A. P. Schmeller D. S. Chatzinotas A. Vredenburg V. T. Loyau A. (2020). Environmental factors and host microbiomes shape host-pathogen dynamics. Trends Parasitol. 36, 616–633. doi: 10.1016/j.pt.2020.04.010, PMID: 32402837

[ref9] Brabin B. Tinto H. Roberts S. A. (2019). Testing an infection model to explain excess risk of preterm birth with long-term iron supplementation in a malaria endemic area. Malar. J. 18:374. doi: 10.1186/s12936-019-3013-6, PMID: 31771607 PMC6880560

[ref10] Cardoso-Jaime V. Dimopoulos G. (2025). *Anopheles gambiae* phagocytic hemocytes promote *plasmodium falciparum* infection by regulating midgut epithelial integrity. Nat. Commun. 16:1465. doi: 10.1038/s41467-025-56313-y, PMID: 39920122 PMC11805967

[ref11] Carlton J. M. (2021). A cornucopia of research resources for the fourth rodent malaria parasite species. BMC Biol. 19:82. doi: 10.1186/s12915-021-01019-y, PMID: 33888109 PMC8063385

[ref12] Chakravarty S. Mandal R. K. Duff M. L. Schmidt N. W. (2019). Intestinal short-chain fatty acid composition does not explain gut microbiota-mediated effects on malaria severity. PLoS One 14:e0214449. doi: 10.1371/journal.pone.0214449, PMID: 30917184 PMC6436795

[ref13] Clevers H. C. Bevins C. L. (2013). Paneth cells: maestros of the small intestinal crypts. Annu. Rev. Physiol. 75, 289–311. doi: 10.1146/annurev-physiol-030212-183744, PMID: 23398152

[ref14] Dada N. Sheth M. Liebman K. Pinto J. Lenhart A. (2018). Whole metagenome sequencing reveals links between mosquito microbiota and insecticide resistance in malaria vectors. Sci. Rep. 8:2084. doi: 10.1038/s41598-018-20367-4, PMID: 29391526 PMC5794770

[ref15] Dehghan H. Oshaghi M. A. Mosa-Kazemi S. H. Abai M. R. Rafie F. Nateghpour M.. (2018). Experimental study on and BALB/c mouse system: implications for malaria transmission blocking assays. Iran. J. Parasitol. 13, 549–559. 30697308 PMC6348208

[ref16] Denny J. E. Powers J. B. Castro H. F. Zhang J. Joshi-Barve S. Campagna S. R.. (2019). Differential sensitivity to *plasmodium yoelii* infection in C57BL/6 mice impacts gut-liver Axis homeostasis. Sci. Rep. 9:3472. doi: 10.1038/s41598-019-40266-6, PMID: 30837607 PMC6401097

[ref17] Drew G. C. Stevens E. J. King K. C. (2021). Microbial evolution and transitions along the parasite-mutualist continuum. Nat. Rev. Microbiol. 19, 623–638. doi: 10.1038/s41579-021-00550-7, PMID: 33875863 PMC8054256

[ref18] Ezenwa V. O. (2016). Helminth-microparasite co-infection in wildlife: lessons from ruminants, rodents and rabbits. Parasite Immunol. 38, 527–534. doi: 10.1111/pim.12348, PMID: 27426017

[ref19] Fackelmann G. Gillingham M. A. F. Schmid J. Heni A. C. Wilhelm K. Schwensow N.. (2021). Human encroachment into wildlife gut microbiomes. Commun. Biol. 4:800. doi: 10.1038/s42003-021-02315-7, PMID: 34172822 PMC8233340

[ref20] Fan Z.-g. Li X. Fu H.-y. Zhou L.-m. Gong F.-l. Fang M. (2019). Gut microbiota reconstruction following host infection with blood-stage *plasmodium berghei* ANKA strain in a murine model. Curr. Med. Sci. 39, 883–889. doi: 10.1007/s11596-019-2119-y, PMID: 31845218

[ref21] Figueiredo M. M. Costa P. A. C. Diniz S. Q. Henriques P. M. Kano F. S. Tada M. S.. (2017). T follicular helper cells regulate the activation of B lymphocytes and antibody production during *plasmodium vivax* infection. PLoS Pathog. 13:e1006484. doi: 10.1371/journal.ppat.1006484, PMID: 28700710 PMC5519210

[ref22] Fishbein S. R. S. Mahmud B. Dantas G. (2023). Antibiotic perturbations to the gut microbiome. Nat. Rev. Microbiol. 21, 772–788. doi: 10.1038/s41579-023-00933-y, PMID: 37491458 PMC12087466

[ref23] Frisan T. (2021). Co-and polymicrobial infections in the gut mucosa: the host-microbiota-pathogen perspective. Cell. Microbiol. 23:e13279. doi: 10.1111/cmi.13279, PMID: 33040471 PMC7900980

[ref24] Georgiadou A. Dunican C. Soro-Barrio P. Lee H. J. Kaforou M. Cunnington A. J. (2022). Comparative transcriptomic analysis reveals translationally relevant processes in mouse models of malaria. eLife 11:e70763. doi: 10.7554/eLife.70763, PMID: 35006075 PMC8747512

[ref25] Gerbe F. Sidot E. Smyth D. J. Ohmoto M. Matsumoto I. Dardalhon V.. (2016). Intestinal epithelial tuft cells initiate type 2 mucosal immunity to helminth parasites. Nature 529, 226–230. doi: 10.1038/nature16527, PMID: 26762460 PMC7614903

[ref26] Guan W. Song X. Yang S. Zhu H. Li F. Li J. (2022). Observation of the gut microbiota profile in BALB/c mice induced by *plasmodium yoelii* 17XL infection. Front. Microbiol. 13:858897. doi: 10.3389/fmicb.2022.858897, PMID: 35432291 PMC9009211

[ref27] Guan W. Xu D. Yang S. Zhao Y. Xie Y. Lin M.. (2023). Observation of intestinal flora diversity with the parasites infection process in a nonlethal malaria model of BALB/c mice induced by *plasmodium yoelii* 17XNL strain. Decod. Infect. Trans. 1:100004. doi: 10.1016/j.dcit.2023.100004

[ref28] Guo J. Song C. Liu Y. Wu X. Dong W. Zhu H.. (2022). Characteristics of gut microbiota in representative mice strains: implications for biological research. Anim. Model Exp. Med. 5, 337–349. doi: 10.1002/ame2.12257, PMID: 35892142 PMC9434578

[ref29] Guo X. Stroup S. E. Houpt E. R. (2008). Persistence of *Entamoeba histolytica* infection in CBA mice owes to intestinal IL-4 production and inhibition of protective IFN-gamma. Mucosal Immunol. 1, 139–146. doi: 10.1038/mi.2007.18, PMID: 19079171

[ref30] Hildebrand F. Nguyen T. L. Brinkman B. Yunta R. G. Cauwe B. Vandenabeele P.. (2013). Inflammation-associated enterotypes, host genotype, cage and inter-individual effects drive gut microbiota variation in common laboratory mice. Genome Biol. 14:R4. doi: 10.1186/gb-2013-14-1-r4, PMID: 23347395 PMC4053703

[ref31] Hoarau A. O. G. Mavingui P. Lebarbenchon C. (2020). Coinfections in wildlife: focus on a neglected aspect of infectious disease epidemiology. PLoS Pathog. 16:e1008790. doi: 10.1371/journal.ppat.1008790, PMID: 32881983 PMC7470396

[ref32] Hou K. Wu Z. X. Chen X. Y. Wang J. Q. Zhang D. Xiao C.. (2022). Microbiota in health and diseases. Signal Transduct. Target. Ther. 7:135. doi: 10.1038/s41392-022-00974-4, PMID: 35461318 PMC9034083

[ref33] Howard G. P. Bender N. G. Khare P. López-Gutiérrez B. Nyasembe V. Weiss W. J.. (2021). Immunopotentiation by lymph-node targeting of a malaria transmission-blocking Nanovaccine. Front. Immunol. 12:729086. doi: 10.3389/fimmu.2021.729086, PMID: 34512663 PMC8432939

[ref34] Howitt M. R. Lavoie S. Michaud M. Blum A. M. Tran S. V. Weinstock J. V.. (2016). Tuft cells, taste-chemosensory cells, orchestrate parasite type 2 immunity in the gut. Science 351, 1329–1333. doi: 10.1126/science.aaf1648, PMID: 26847546 PMC5528851

[ref35] Huang J. Li X. Coelho-dos-Reis J. G. Zhang M. Mitchell R. Nogueira R. T.. (2015). Human immune system mice immunized with *plasmodium falciparum* circumsporozoite protein induce protective human humoral immunity against malaria. J. Immunol. Methods 427, 42–50. doi: 10.1016/j.jim.2015.09.005, PMID: 26410104

[ref36] Hugenholtz F. de Vos W. M. (2018). Mouse models for human intestinal microbiota research: a critical evaluation. Cell. Mol. Life Sci. 75, 149–160. doi: 10.1007/s00018-017-2693-8, PMID: 29124307 PMC5752736

[ref37] Ianiro G. Maida M. Burisch J. Simonelli C. Hold G. Ventimiglia M.. (2018). Efficacy of different faecal microbiota transplantation protocols for *Clostridium difficile* infection: a systematic review and meta-analysis. United European Gastroenterol J 6, 1232–1244. doi: 10.1177/2050640618780762, PMID: 30288286 PMC6169051

[ref38] Ippolito M. M. Denny J. E. Langelier C. Sears C. L. Schmidt N. W. (2018). Malaria and the microbiome: a systematic review. Clin. Infect. Dis. 67, 1831–1839. doi: 10.1093/cid/ciy374, PMID: 29701835 PMC6260159

[ref39] Ivanov I. I. Tuganbaev T. Skelly A. N. Honda K. (2022). T cell responses to the microbiota. Annu. Rev. Immunol. 40, 559–587. doi: 10.1146/annurev-immunol-101320-011829, PMID: 35113732 PMC9296687

[ref40] Ivory C. P. Prystajecky M. Jobin C. Chadee K. (2008). Toll-like receptor 9-dependent macrophage activation by *Entamoeba histolytica* DNA. Infect. Immun. 76, 289–297. doi: 10.1128/iai.01217-07, PMID: 17984204 PMC2223673

[ref41] Kamada N. Chen G. Y. Inohara N. Núñez G. (2013). Control of pathogens and pathobionts by the gut microbiota. Nat. Immunol. 14, 685–690. doi: 10.1038/ni.2608, PMID: 23778796 PMC4083503

[ref42] Khosravi A. Mazmanian S. K. (2013). Disruption of the gut microbiome as a risk factor for microbial infections. Curr. Opin. Microbiol. 16, 221–227. doi: 10.1016/j.mib.2013.03.009, PMID: 23597788 PMC5695238

[ref43] Kim M. H. Kang S. G. Park J. H. Yanagisawa M. Kim C. H. (2013). Short-chain fatty acids activate GPR41 and GPR43 on intestinal epithelial cells to promote inflammatory responses in mice. Gastroenterology 145:396-406.e391-310. doi: 10.1053/j.gastro.2013.04.056, PMID: 23665276

[ref44] Knowler S. A. Shindler A. Wood J. L. Lakkavaram A. Thomas C. J. de Koning-Ward T. F.. (2023). Altered gastrointestinal tract structure and microbiome following cerebral malaria infection. Parasitol. Res. 122, 789–799. doi: 10.1007/s00436-022-07775-2, PMID: 36602586

[ref45] Kohl K. D. Weiss R. B. Cox J. Dale C. Dearing M. D. (2014). Gut microbes of mammalian herbivores facilitate intake of plant toxins. Ecol. Lett. 17, 1238–1246. doi: 10.1111/ele.12329, PMID: 25040855

[ref46] Krishnan J. (2017). Human cerebral malaria and experimental cerebral malaria in mice: relevance and applicability. MOJ Biol. Med. 2, 186–187. doi: 10.15406/mojbm.2017.02.00041

[ref47] Krych L. Hansen C. H. Hansen A. K. van den Berg F. W. Nielsen D. S. (2013). Quantitatively different, yet qualitatively alike: a meta-analysis of the mouse core gut microbiome with a view towards the human gut microbiome. PLoS One 8:e62578. doi: 10.1371/journal.pone.0062578 23658749 PMC3641060

[ref48] Li X. Huang J. Zhang M. Funakoshi R. Sheetij D. Spaccapelo R.. (2016). Human CD8+ T cells mediate protective immunity induced by a human malaria vaccine in human immune system mice. Vaccine 34, 4501–4506. doi: 10.1016/j.vaccine.2016.08.006, PMID: 27502569 PMC5009892

[ref49] Ling Z. Liu X. Jia X. Cheng Y. Luo Y. Yuan L.. (2014). Impacts of infection with different toxigenic *Clostridium difficile* strains on faecal microbiota in children. Sci. Rep. 4:7485. doi: 10.1038/srep07485, PMID: 25501371 PMC4265774

[ref50] Lotter H. González-Roldán N. Lindner B. Winau F. Isibasi A. Moreno-Lafont M.. (2009). Natural killer T cells activated by a lipopeptidophosphoglycan from *Entamoeba histolytica* are critically important to control amebic liver abscess. PLoS Pathog. 5:e1000434. doi: 10.1371/journal.ppat.1000434, PMID: 19436711 PMC2674934

[ref51] Maizels R. M. Hewitson J. P. Smith K. A. (2012). Susceptibility and immunity to helminth parasites. Curr. Opin. Immunol. 24, 459–466. doi: 10.1016/j.coi.2012.06.003, PMID: 22795966 PMC3437973

[ref52] Mancio-Silva L. Slavic K. Grilo Ruivo M. T. Grosso A. R. Modrzynska K. K. Vera I. M.. (2017). Nutrient sensing modulates malaria parasite virulence. Nature 547, 213–216. doi: 10.1038/nature23009, PMID: 28678779 PMC5511512

[ref53] Mandal R. K. Denny J. E. Waide M. L. Li Q. Bhutiani N. Anderson C. D.. (2020). Temporospatial shifts within commercial laboratory mouse gut microbiota impact experimental reproducibility. BMC Biol. 18:83. doi: 10.1186/s12915-020-00810-7, PMID: 32620114 PMC7334859

[ref54] Mandal R. K. Mandal A. Denny J. E. Namazii R. John C. C. Schmidt N. W. (2023). Gut Bacteroides act in a microbial consortium to cause susceptibility to severe malaria. Nat. Commun. 14:6465. doi: 10.1038/s41467-023-42235-0, PMID: 37833304 PMC10575898

[ref55] McCauley H. A. Guasch G. (2015). Three cheers for the goblet cell: maintaining homeostasis in mucosal epithelia. Trends Mol. Med. 21, 492–503. doi: 10.1016/j.molmed.2015.06.003, PMID: 26144290

[ref56] Mehdi S. F. Qureshi M. H. Pervaiz S. Kumari K. Saji E. Shah M.. (2025). Endocrine and metabolic alterations in response to systemic inflammation and sepsis: a review article. Mol. Med. 31:16. doi: 10.1186/s10020-025-01074-z, PMID: 39838305 PMC11752782

[ref57] Mikolajczak S. A. Vaughan A. M. Kangwanrangsan N. Roobsoong W. Fishbaugher M. Yimamnuaychok N.. (2015). Plasmodium vivax liver stage development and hypnozoite persistence in human liver-chimeric mice. Cell Host Microbe 17, 526–535. doi: 10.1016/j.chom.2015.02.011, PMID: 25800544 PMC5299596

[ref58] Minkah N. K. Schafer C. Kappe S. H. I. (2018). Humanized mouse models for the study of human malaria parasite biology, pathogenesis, and immunity. Front. Immunol. 9:807. doi: 10.3389/fimmu.2018.00807, PMID: 29725334 PMC5917005

[ref59] Mooney J. P. Lokken K. L. Byndloss M. X. George M. D. Velazquez E. M. Faber F.. (2015). Inflammation-associated alterations to the intestinal microbiota reduce colonization resistance against non-typhoidal Salmonella during concurrent malaria parasite infection. Sci. Rep. 5:14603. doi: 10.1038/srep14603, PMID: 26434367 PMC4592952

[ref60] Morffy Smith C. D. Gong M. Andrew A. K. Russ B. N. Ge Y. Zadeh M.. (2019). Composition of the gut microbiota transcends genetic determinants of malaria infection severity and influences pregnancy outcome. EBioMedicine 44, 639–655. doi: 10.1016/j.ebiom.2019.05.052, PMID: 31160271 PMC6606560

[ref61] Mukherjee D. Chora Â. F. Lone J.-C. Ramiro R. S. Blankenhaus B. Serre K.. (2022). Host lung microbiota promotes malaria-associated acute respiratory distress syndrome. Nat. Commun. 13:3747. doi: 10.1038/s41467-022-31301-8, PMID: 35768411 PMC9243033

[ref62] Murr N. J. Olender T. B. Smith M. R. Smith A. S. Pilotos J. Richard L. B.. (2021). *Plasmodium chabaudi* infection alters intestinal morphology and mucosal innate immunity in moderately malnourished mice. Nutrients 13:913. doi: 10.3390/nu13030913, PMID: 33799736 PMC7998862

[ref63] Neurath M. F. Artis D. Becker C. (2025). The intestinal barrier: a pivotal role in health, inflammation, and cancer. Lancet Gastroenterol. Hepatol. 10, 573–592. doi: 10.1016/s2468-1253(24)00390-x, PMID: 40086468

[ref64] Nguyen T. L. Vieira-Silva S. Liston A. Raes J. (2015). How informative is the mouse for human gut microbiota research? Dis. Model. Mech. 8, 1–16. doi: 10.1242/dmm.017400, PMID: 25561744 PMC4283646

[ref65] Panzer A. R. Lynch S. V. (2015). Influence and effect of the human microbiome in allergy and asthma. Curr. Opin. Rheumatol. 27, 373–380. doi: 10.1097/bor.0000000000000191, PMID: 26002029

[ref66] Pattaradilokrat S. Wu J. Xu F. Su X. Z. (2022). The origins, isolation, and biological characterization of rodent malaria parasites. Parasitol. Int. 91:102636. doi: 10.1016/j.parint.2022.102636, PMID: 35926694 PMC9465976

[ref67] Pérez-Mazliah D. Ng D. H. Freitas do Rosário A. P. McLaughlin S. Mastelic-Gavillet B. Sodenkamp J.. (2015). Disruption of IL-21 signaling affects T cell-B cell interactions and abrogates protective humoral immunity to malaria. PLoS Pathog. 11:e1004715. doi: 10.1371/journal.ppat.1004715, PMID: 25763578 PMC4370355

[ref68] Pérez-Mazliah D. Nguyen M. P. Hosking C. McLaughlin S. Lewis M. D. Tumwine I.. (2017). Follicular helper T cells are essential for the elimination of *plasmodium* infection. EBioMedicine 24, 216–230. doi: 10.1016/j.ebiom.2017.08.030, PMID: 28888925 PMC5652023

[ref69] Quraishi M. N. Widlak M. Bhala N. Moore D. Price M. Sharma N.. (2017). Systematic review with meta-analysis: the efficacy of faecal microbiota transplantation for the treatment of recurrent and refractory *Clostridium difficile* infection. Aliment. Pharmacol. Ther. 46, 479–493. doi: 10.1111/apt.14201, PMID: 28707337

[ref70] Ramírez-Carrillo E. Gaona O. Nieto J. Sánchez-Quinto A. Cerqueda-García D. Falcón L. I.. (2020). Disturbance in human gut microbiota networks by parasites and its implications in the incidence of depression. Sci. Rep. 10:3680. doi: 10.1038/s41598-020-60562-w, PMID: 32111922 PMC7048763

[ref71] Rosa-Fernandes L. Santiago V. F. Silva-Santos Y. D. Lopes T. T. Peixoto E. P. M. Rodrigues S. A. M.. (2025). Serum proteomics of experimental malaria-associated ARDS reveals a regulation of acute-phase response proteins. J Immunol Res 2025:5642957. doi: 10.1155/jimr/5642957 40160901 PMC11955258

[ref72] Shaw M. H. Reimer T. Sánchez-Valdepeñas C. Warner N. Kim Y. G. Fresno M.. (2009). T cell-intrinsic role of Nod2 in promoting type 1 immunity to toxoplasma gondii. Nat. Immunol. 10, 1267–1274. doi: 10.1038/ni.1816, PMID: 19881508 PMC2803073

[ref73] Soulard V. Bosson-Vanga H. Lorthiois A. Roucher C. Franetich J. F. Zanghi G.. (2015). Plasmodium falciparum full life cycle and plasmodium ovale liver stages in humanized mice. Nat. Commun. 6:7690. doi: 10.1038/ncomms8690, PMID: 26205537 PMC4525212

[ref74] Spragge F. Bakkeren E. Jahn M. T. E B. N. A. Pearson C. F. Wang X.. (2023). Microbiome diversity protects against pathogens by nutrient blocking. Science 382:eadj3502. doi: 10.1126/science.adj3502 38096285 PMC7616675

[ref75] Sriboonvorakul N. Chotivanich K. Silachamroon U. Phumratanaprapin W. Adams J. H. Dondorp A. M.. (2023). Intestinal injury and the gut microbiota in patients with *plasmodium falciparum* malaria. PLoS Pathog. 19:e1011661. doi: 10.1371/journal.ppat.1011661, PMID: 37856470 PMC10586672

[ref76] Staley C. Kelly C. R. Brandt L. J. Khoruts A. Sadowsky M. J. (2016). Complete microbiota engraftment is not essential for recovery from recurrent *Clostridium difficile* infection following fecal microbiota transplantation. MBio 7:e01965-16. doi: 10.1128/mBio.01965-16, PMID: 27999162 PMC5181777

[ref77] Stecher B. Maier L. Hardt W. D. (2013). 'Blooming' in the gut: how dysbiosis might contribute to pathogen evolution. Nat. Rev. Microbiol. 11, 277–284. doi: 10.1038/nrmicro2989, PMID: 23474681

[ref78] Stefka A. T. Feehley T. Tripathi P. Qiu J. McCoy K. Mazmanian S. K.. (2014). Commensal bacteria protect against food allergen sensitization. Proc. Natl. Acad. Sci. USA 111, 13145–13150. doi: 10.1073/pnas.1412008111, PMID: 25157157 PMC4246970

[ref79] Stephens R. Culleton R. L. Lamb T. J. (2012). The contribution of *plasmodium chabaudi* to our understanding of malaria. Trends Parasitol. 28, 73–82. doi: 10.1016/j.pt.2011.10.006, PMID: 22100995 PMC4040349

[ref80] Stoltzfus R. J. (2012). Iron and malaria interactions: programmatic ways forward. Adv. Nutr. 3, 579–582. doi: 10.3945/an.111.000885, PMID: 22797995 PMC3649729

[ref81] Stough J. M. Dearth S. P. Denny J. E. LeCleir G. R. Schmidt N. W. Campagna S. R.. (2016). Functional characteristics of the gut microbiome in C57BL/6 mice differentially susceptible to *plasmodium yoelii*. Front. Microbiol. 7:1520. doi: 10.3389/fmicb.2016.01520, PMID: 27729904 PMC5037233

[ref82] Suez J. Zmora N. Segal E. Elinav E. (2019). The pros, cons, and many unknowns of probiotics. Nat. Med. 25, 716–729. doi: 10.1038/s41591-019-0439-x, PMID: 31061539

[ref83] Swardson-Olver C. J. Dawson T. C. Burnett R. C. Peiper S. C. Maeda N. Avery A. C. (2002). *Plasmodium yoelii* uses the murine Duffy antigen receptor for chemokines as a receptor for normocyte invasion and an alternative receptor for reticulocyte invasion. Blood 99, 2677–2684. doi: 10.1182/blood.v99.8.2677, PMID: 11929753

[ref84] Takeuchi T. Kubota T. Nakanishi Y. Tsugawa H. Suda W. Kwon A. T.. (2023). Gut microbial carbohydrate metabolism contributes to insulin resistance. Nature 621, 389–395. doi: 10.1038/s41586-023-06466-x, PMID: 37648852 PMC10499599

[ref85] Taniguchi T. Miyauchi E. Nakamura S. Hirai M. Suzue K. Imai T.. (2015). *Plasmodium berghei* ANKA causes intestinal malaria associated with dysbiosis. Sci. Rep. 5:15699. doi: 10.1038/srep15699, PMID: 26503461 PMC4621605

[ref86] Teng F. Klinger C. N. Felix K. M. Bradley C. P. Wu E. Tran N. L.. (2016). Gut microbiota drive autoimmune arthritis by promoting differentiation and migration of Peyer's patch T follicular helper cells. Immunity 44, 875–888. doi: 10.1016/j.immuni.2016.03.013, PMID: 27096318 PMC5296410

[ref87] Tripathi A. Debelius J. Brenner D. A. Karin M. Loomba R. Schnabl B.. (2018). The gut-liver axis and the intersection with the microbiome. Nat. Rev. Gastroenterol. Hepatol. 15, 397–411. doi: 10.1038/s41575-018-0011-z, PMID: 29748586 PMC6319369

[ref88] Vaughan A. M. Pinapati R. S. Cheeseman I. H. Camargo N. Fishbaugher M. Checkley L. A.. (2015). Plasmodium falciparum genetic crosses in a humanized mouse model. Nat. Methods 12, 631–633. doi: 10.1038/nmeth.3432, PMID: 26030447 PMC4547688

[ref89] Villarino N. F. LeCleir G. R. Denny J. E. Dearth S. P. Harding C. L. Sloan S. S.. (2016). Composition of the gut microbiota modulates the severity of malaria. Proc. Natl. Acad. Sci. USA 113, 2235–2240. doi: 10.1073/pnas.1504887113, PMID: 26858424 PMC4776451

[ref90] Vinayagam S. Rajendran D. Sekar K. Renu K. Sattu K. (2023). The microbiota, the malarial parasite, and the mosquito [MMM] - a three-sided relationship. Mol. Biochem. Parasitol. 253:111543. doi: 10.1016/j.molbiopara.2023.111543, PMID: 36642385

[ref91] Vivanco-Cid H. Alpuche-Aranda C. Wong-Baeza I. Rocha-Ramírez L. M. Rios-Sarabia N. Estrada-Garcia I.. (2007). Lipopopeptidephosphoglycan from *Entamoeba histolytica* activates human macrophages and dendritic cells and reaches their late endosomes. Parasite Immunol. 29, 467–474. doi: 10.1111/j.1365-3024.2007.00963.x, PMID: 17727570

[ref92] Waide M. L. Polidoro R. Powell W. L. Denny J. E. Kos J. Tieri D. A.. (2020). Gut microbiota composition modulates the magnitude and quality of germinal centers during *plasmodium* infections. Cell Rep. 33:108503. doi: 10.1016/j.celrep.2020.108503, PMID: 33326773 PMC7772993

[ref93] Waide M. L. Schmidt N. W. (2020). The gut microbiome, immunity, and *plasmodium* severity. Curr. Opin. Microbiol. 58, 56–61. doi: 10.1016/j.mib.2020.08.006, PMID: 33007644 PMC7746623

[ref94] Walker G. T. Yang G. Tsai J. Y. Rodriguez J. L. English B. C. Faber F.. (2021). Malaria parasite infection compromises colonization resistance to an enteric pathogen by reducing gastric acidity. Sci. Adv. 7:eabd6232. doi: 10.1126/sciadv.abd6232, PMID: 34193410 PMC8245046

[ref95] White N. J. Turner G. D. Medana I. M. Dondorp A. M. Day N. P. (2010). The murine cerebral malaria phenomenon. Trends Parasitol. 26, 11–15. doi: 10.1016/j.pt.2009.10.007, PMID: 19932638 PMC2807032

[ref96] WHO (2024). World Malaria Report 2024. Geneva: WHO Press.

[ref97] Wilson I. D. Nicholson J. K. (2017). Gut microbiome interactions with drug metabolism, efficacy, and toxicity. Transl. Res. 179, 204–222. doi: 10.1016/j.trsl.2016.08.002, PMID: 27591027 PMC5718288

[ref98] Wotzka S. Y. Nguyen B. D. Hardt W. D. (2017). *Salmonella Typhimurium* diarrhea reveals basic principles of Enteropathogen infection and disease-promoted DNA exchange. Cell Host Microbe 21, 443–454. doi: 10.1016/j.chom.2017.03.009, PMID: 28407482

[ref99] Wright J. K. Weckman A. M. Ngai M. Stefanova V. Zhong K. McDonald C. R.. (2023). Intestinal barrier disruption with *plasmodium falciparum* infection in pregnancy and risk of preterm birth: a cohort study. EBioMedicine 97:104808. doi: 10.1016/j.ebiom.2023.104808, PMID: 37837932 PMC10585225

[ref100] Xiao L. Feng Q. Liang S. Sonne S. B. Xia Z. Qiu X.. (2015). A catalog of the mouse gut metagenome. Nat. Biotechnol. 33, 1103–1108. doi: 10.1038/nbt.3353, PMID: 26414350

[ref101] Yarovinsky F. (2014). Innate immunity to toxoplasma gondii infection. Nat. Rev. Immunol. 14, 109–121. doi: 10.1038/nri3598, PMID: 24457485

[ref102] Yawen Z. Xiangyun C. Binyou L. Xingchen Y. Taiping L. Xuedong Z.. (2022). The dynamic landscape of parasitemia dependent intestinal microbiota shifting and the correlated gut transcriptome during *plasmodium yoelii* infection. Microbiol. Res. 258:126994. doi: 10.1016/j.micres.2022.126994, PMID: 35220138

[ref103] Yilmaz B. Li H. (2018). Gut microbiota and Iron: the crucial actors in health and disease. Pharmaceuticals 11:98. doi: 10.3390/ph11040098, PMID: 30301142 PMC6315993

[ref104] Yilmaz B. Portugal S. Tran T. M. Gozzelino R. Ramos S. Gomes J.. (2014). Gut microbiota elicits a protective immune response against malaria transmission. Cell 159, 1277–1289. doi: 10.1016/j.cell.2014.10.053, PMID: 25480293 PMC4261137

[ref105] Yoo J. Y. Groer M. Dutra S. V. O. Sarkar A. McSkimming D. I. (2020). Gut microbiota and immune system interactions. Microorganisms 8:1587. doi: 10.3390/microorganisms8101587, PMID: 33076307 PMC7602490

[ref106] Yooseph S. Kirkness E. F. Tran T. M. Harkins D. M. Jones M. B. Torralba M. G.. (2015). Stool microbiota composition is associated with the prospective risk of *plasmodium falciparum* infection. BMC Genomics 16:631. doi: 10.1186/s12864-015-1819-3, PMID: 26296559 PMC4546150

[ref107] Zhang P. Zhao X. Wang Y. Du K. Wang Z. Yu J.. (2022). Bacteriophage protein Gp46 is a cross-species inhibitor of nucleoid-associated HU proteins. Proc. Natl. Acad. Sci. USA 119:e2116278119. doi: 10.1073/pnas.2116278119, PMID: 35193978 PMC8892312

[ref108] Zuzarte-Luís V. Mello-Vieira J. Marreiros I. M. Liehl P. Chora Â F. Carret C. K.. (2017). Dietary alterations modulate susceptibility to *plasmodium* infection. Nat. Microbiol. 2, 1600–1607. doi: 10.1038/s41564-017-0025-2 28947801

